# Nanophotonic device design based on large language models: multilayer and metasurface examples

**DOI:** 10.1515/nanoph-2024-0674

**Published:** 2025-02-17

**Authors:** Myungjoon Kim, Hyeonjin Park, Jonghwa Shin

**Affiliations:** KAIST, Daejeon, Republic of Korea

**Keywords:** large language model, inverse design, in-context learning, fine-tuning

## Abstract

Large language models (LLMs) have gained significant prominence in language processing, demonstrating remarkable performance across a wide range of tasks. Recently, LLMs have been explored in various scientific fields beyond language-based tasks. However, their application in the design of nanophotonic devices remains less explored. Here, we investigate the capabilities of LLMs to address nanophotonic design problems without requiring domain-specific expertise of the user. Our findings show that an LLM with in-context learning enables nonexpert users to calculate optical responses of multilayer films via numerical simulations. Through conversational interaction and feedback between the LLM and the user, an optimal design of the multilayer films can be also produced for the user-provided target optical properties. Furthermore, we fine-tune the LLM using text-based representations of the structure and properties of optical metasurfaces. We demonstrate that the fine-tuned LLM can generate metasurface designs with target properties by reversing the input and output text. This research highlights the potential of LLMs to expedite the nanophotonic design process and to make it more accessible to a wider audience.

## 1 Introduction

In recent decades, the field of nanophotonics has seen significant advancements, driven by continuous improvements in nanofabrication capabilities. This progress necessitates a versatile design approach for nanophotonic devices, where the shapes, sizes, and positions of different nanostructures are properly optimized to control light scattering in desired ways. The vast design spaces render the trial-and-error approach impractical in most cases, and various optimization algorithms have been developed, enabling efficient design explorations [1], [2], [3]. Building on these advances, there has been strong interest in “inverse design” methods, which can drastically reduce the design iterations needed to produce the optimal design for given desired optical properties. A popular approach is gradient-based optimization, which, when combined with the adjoint simulations, allows rapid gradient calculations [4] and efficient iterative updates of performance. Inverse design has been extensively applied in a variety of photonic devices, including lenses [5], [6], beam deflectors [7], lithography [8], [9], and optical analog computing devices [10], [11], [12].

Although inverse design has proven effective, it requires detailed knowledge of photonic systems and complex computational algorithms, demanding a solid foundation in optics and optimization from the researchers. A typical design process involves (i) setting up simulations correctly to ensure accuracy of the results, (ii) defining objective functions that properly reflect device performance, and (iii) choosing a well-suited optimization algorithm and adjusting their hyperparameters properly, before the actual optimization can start. These tasks present a significant hurdle for nonexperts due to the need for specialized knowledge and skill. While graphic user interface-based commercial software with built-in optimization routines can lower some of the initial hurdles, it still requires significant learning time for correct usage. As a result, nanophotonic design tasks have been reserved for those who can invest many hours and concentrated effort for the job.

What makes the design process even less accessible is the fact that obtaining the optical properties of general nanophotonic systems such as metasurfaces and photonic integrated circuits usually requires full-wave vectorial simulations, which are often computationally intensive necessitating dedicated large-scale computing resource. To address this challenge, data-driven surrogate models have emerged, providing highly accurate results with significantly reduced computational demands while effectively addressing various nanophotonic structures and properties [13], [14], [15], [16], [17], [18]. These models can predict the structure–performance relation in a much shorter time or allow use of lighter resources, albeit with some decrease in prediction accuracy. Performance of the model can be further enhanced by considering underlying physics and adapting the architecture of the surrogate model to domain-specific knowledge. For instance, WaveYNet [17] uses a modified U-net architecture to meet the physics-specific demands of photonic systems, including the appropriate representation of inputs and outputs, boundary-aware padding, and a physics-informed loss function. However, developing a machine learning model tailored to nanophotonic systems requires expertise in both artificial neural networks and physics, along with extensive training for hyperparameter optimization. When the photonic system changes, it may be necessary to develop a new neural network model to meet different physical requirements. It presents an additional hurdle due to the specialized knowledge required.

In this study, we demonstrate that large language models (LLMs) can help address many of the challenges mentioned above, enabling the design of nanophotonic devices without requiring domain-specific expertise. LLMs are capable of comprehending nanophotonic systems and performing design optimization through two advanced techniques. First, we employ in-context learning (ICL), a technique that allows LLMs to interpret tasks without further training, to generate code for calculating the spectral response of multilayer films. By incorporating iterative prompting with feedback, this approach also enables optimal design of multilayer films to achieve target optical functionalities. Second, we examine that fine-tuning the LLM with a text-encoded nanophotonic dataset allows it to predict the spectral responses of all-dielectric metasurfaces. Additionally, by encoding property-to-structure relationships, the fine-tuned LLM can generate metasurface structures with desired spectral properties.

This paper is organized as follows. Section 2 provides background knowledge on LLMs and introduces the related work in photonic design. In Section 3, we explain the application of ICL to the design of multilayer films. Section 4 introduces the fine-tuning approach for forward prediction and inverse design of all-dielectric metasurfaces. Finally, Section 5 concludes the paper by summarizing the key findings and exploring potential future applications of LLMs in nanophotonic design.

## 2 Large language models

Language models are artificial intelligence (AI) systems built to understand and generate natural-language text across diverse subject fields. These models take sequences of text as input and, by analyzing their structure and context, generate relevant output sequence of words that align with the given input. LLMs, language models with billions or more parameters, have recently gained substantial attention for their exceptional capabilities in language-based applications [19].

The impressive capabilities of LLMs can be attributed to three key factors within transformer architecture. First, the transformer architecture utilizes self-attention mechanisms [20], allowing the model to assign varying degrees of importance to different words in a sentence, thereby enhancing its ability to manage long-range dependencies. Second, transformers allow for efficient parallelization, greatly accelerating computations. Finally, transformer architectures demonstrate the scaling hypothesis [21], which suggests that increasing the number of parameters, dataset size, and computational power significantly enhances model performance.

**Figure 1:**
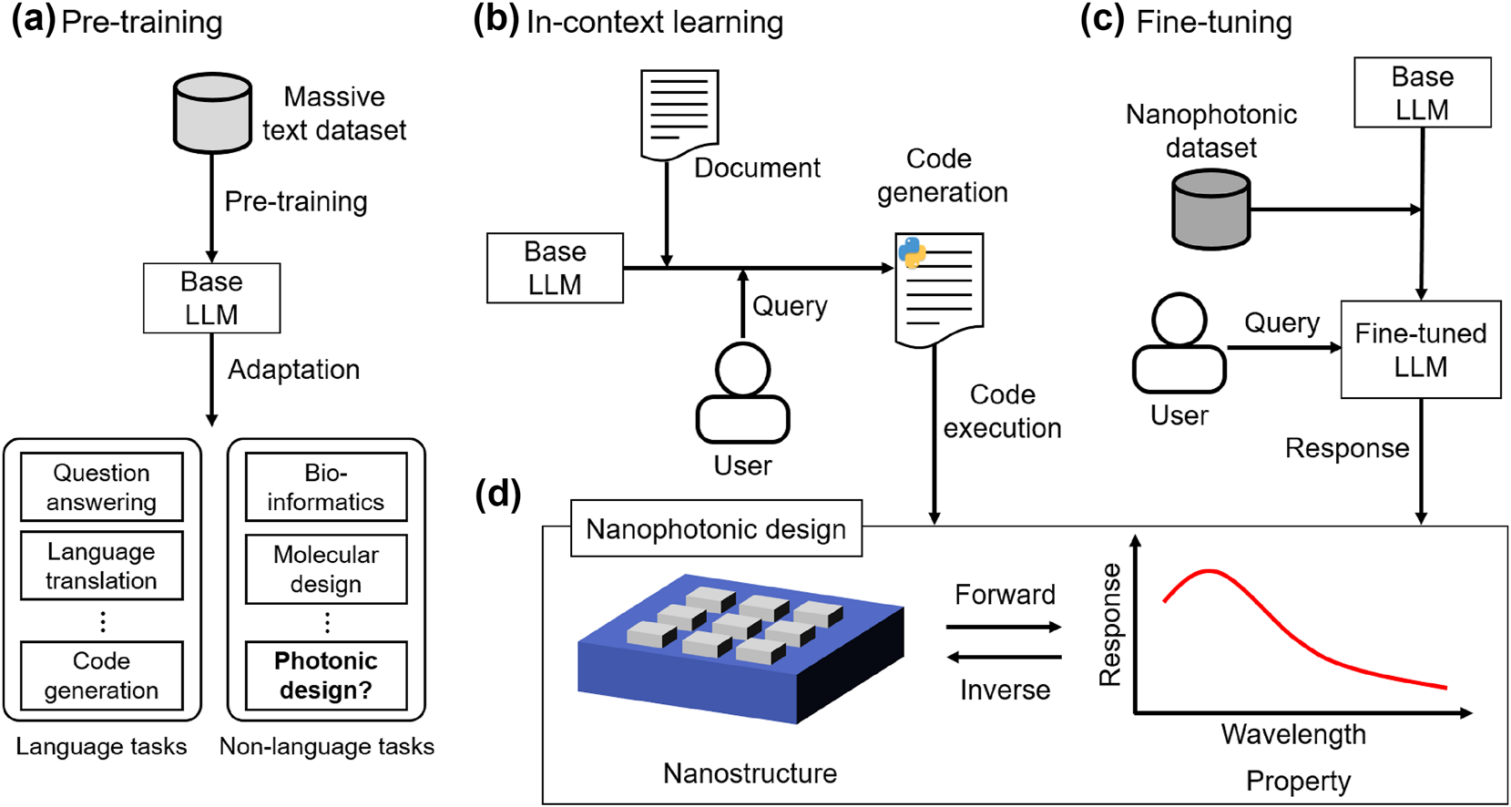
Schematic illustrations of large language models applied to nanophotonic design. (a) Pretraining on extensive text datasets forms a base LLM capable of rapid adaptation to both language and nonlanguage tasks. (b) In-context learning with advanced prompts enables code generation for forward modeling and conversational design. (c) Fine-tuning with nanophotonic-specific datasets facilitates property prediction and inverse design.

The model’s general language understanding is achieved through pretraining. Figure 1a illustrates the process of training LLMs for downstream applications. In this pretraining phase, the model learns to predict the next word in a sequence, enabling it to understand natural language from a massive text dataset. Following pretraining, the model is adapted to perform specific tasks. Through these steps, recent language models have attained expert-level performance across a variety of language tasks, including question answering, language translation, and code generation. They have also been successfully applied to nonlanguage tasks such as bioinformatics and molecular design. However, a natural question arises as to whether LLMs can be effectively applied to the field of photonic design.

To address this question, it is essential to explore various methods that can further enhance LLM functionality. One such technique is ICL, where the model interprets tasks directly from the input prompt, enabling it to perform problem-specific downstream tasks without further training [22], [23], [24], [25]. ICL is performed through prompt engineering, with techniques such as chain-of-thought (CoT) prompting [26] and few-shot prompting [22]. CoT facilitates complex reasoning by prompting the model to break down problems into sequential, intermediate steps, helping it arrive at more accurate and interpretable responses, especially for tasks that benefit from multistep reasoning. Few-shot prompting, on the other hand, improves task comprehension by presenting the model with a small set of exemplary input–output pairs. These examples help the model infer the desired task format and output style, allowing it to generalize better to new, similar inputs even without task-specific training.

Fine-tuning is another approach to enhance LLM functionality, where a pretrained model’s weights are adjusted via supervised learning on a domain-specific dataset. This process allows LLMs to effectively transition from general language understanding to more specific, targeted tasks. A key advantage of fine-tuning in the context of LLMs is that, regardless of the task, the model learns through next-word prediction, enabling flexible adaptation to a wide range of downstream tasks [27]. While effective for these applications, full fine-tuning can be computationally intensive due to the large size of the models. To address this, parameter-efficient fine-tuning methods have been developed. A popular technique, Low-rank Adaptation (LoRA) [28], involves attaching adapters with a small set of parameters using low-rank decomposition matrices while keeping the original pretrained weights frozen. These adapters are added to each self-attention and feedforward layer, significantly reducing the number of trainable parameters and memory requirements.

Recent studies have explored the potential of using transformers and LLMs for optical design tasks. For example, OptoGPT [29], a decoder-only transformer architecture, has been specifically tailored to design multilayer films. Trained on a large dataset containing various multilayer film configurations, OptoGPT can design across a range of target specifications and different multilayer structures, leveraging a single foundation model. In a separate line of research, LLMs such as GPT-3.5 have been adapted to predict metasurface properties by fine-tuning [30]. However, it remains to be seen whether LLMs can be fine-tuned explicitly for inverse design tasks and directly produce the optimal design without design iterations.

This work takes inspiration from these studies, demonstrating that photonic design can be effectively achieved through both prompt engineering with domain-specific text and fine-tuning on a language-interfaced nanophotonic design dataset. By leveraging LLMs to grasp the underlying physics of photonic systems, generate calculation code, and directly infer outputs, we achieve nanophotonic design with desired properties while minimizing the need for extensive expertise in machine learning or optics, making the approach broadly accessible.

## 3 In-context learning

**Figure 2:**
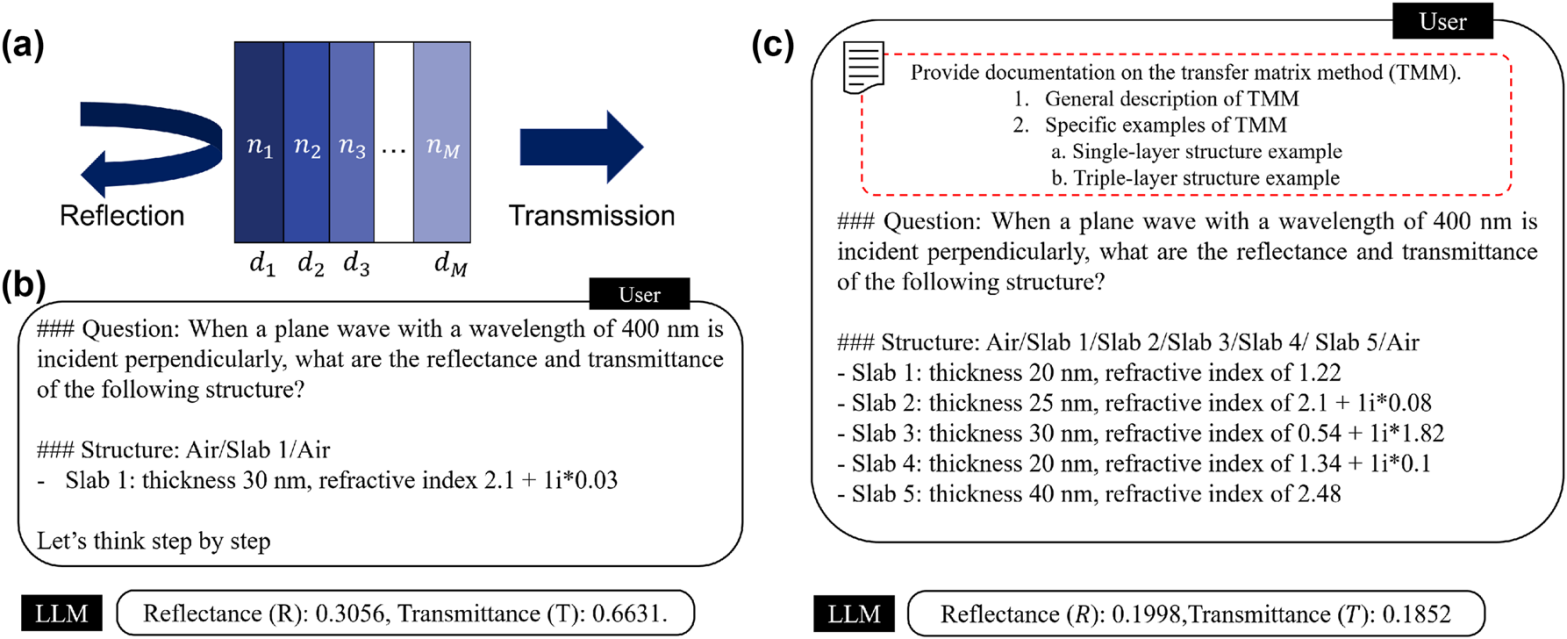
Forward modeling of multilayer films using LLM. (a) Schematic of the multilayer structure. Reflection and transmission of the structure are determined by the thickness and refractive index distribution. (b) Example of a zero-shot CoT prompt and response. (c) Example of a few-shot CoT prompt and response. In this case, the few-shot prompting includes a more detailed lecture note compared to the one-shot scenario. The lecture note offers a step-by-step general explanation along with two specific example problems related to the transfer matrix method. The language model effectively utilizes the information from the lecture note to accurately perform both inference and code generation tasks.

In this section, we explore the effectiveness of in-context learning through prompt engineering to guide LLMs in the modeling and design of photonic nanostructures, even when the users themselves are not experts in optics or physics in general. Our focus is on multilayer optical films, which are composed of thin layers with varying refractive indices and thicknesses, as illustrated in Figure 2a. The spectral properties of these films are determined by their structural variations, making them suitable for a range of optical applications [31], [32], [33], [34]. The wave propagation in multilayer structures can be accurately calculated by the transfer matrix method (TMM), an approach that utilizes matrices to represent the optical behavior of each layer. We assume that the user does not have prior knowledge in optics but have an access to a lecture note on TMM. A primary design challenge lies in optimizing the refractive index and thickness of each layer to achieve the desired spectral properties. All ICL experiments were conducted using the OpenAI ChatGPT-4o model.

### 3.1 Forward modeling of multilayer films

**Figure 3:**
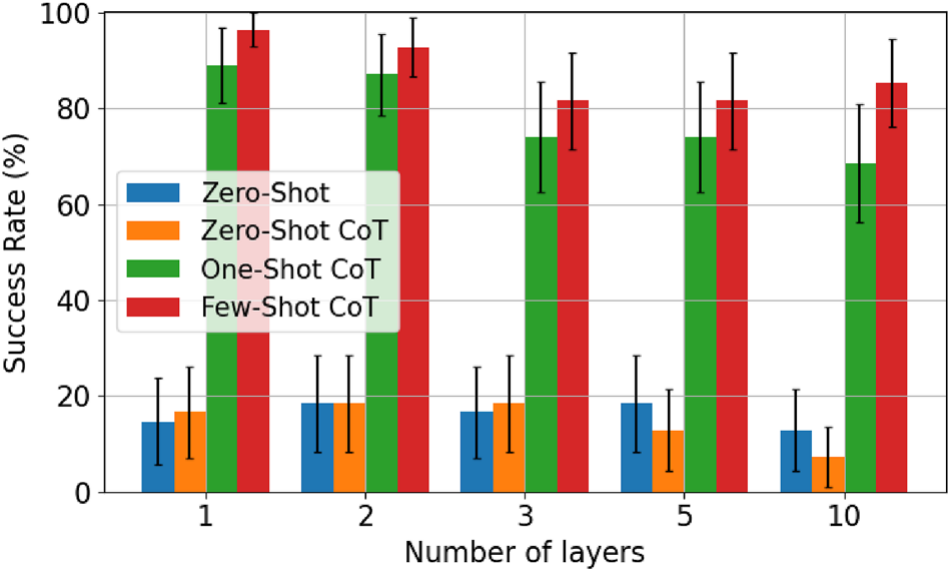
Benchmark results for numerical simulation of multilayer films using code generated by ChatGPT. We report the average success rates obtained across 50 trials for each of the four different prompt engineering techniques. The error bars represent the 95 % confidence intervals. Experiment conducted on January 15, 2025.

In the forward modeling task, we utilized LLM’s capabilities to generate codes to calculate the transmittance and reflectance of multilayer structures. Benchmarks were conducted on five configurations: one, two, three, five, and finally 10 layers. Four distinct prompting techniques were tested, recording the number of successful calculations in 50 trials for each technique. A calculation was considered successful if all steps and output values were correct, and these results were used to evaluate the success rate of each approach. The benchmark results are summarized in Figure 3, and full details of the experiments can be found in the Supplementary materials.

We initially tested zero-shot prompting without providing the lecture note. We found that the LLM failed to generate the correct code for the spectral responses in all five cases. This failure was mainly due to the generated code containing equations that were inconsistent with the principles of the TMM calculations (see Supplementary materials Figure S4). Although the LLM-produced code could execute and generate results, the outputs deviated significantly from the expected values.

Building on this initial attempt, we next tested a zero-shot CoT approach. As shown in Figure 2b, this method added the phrase “Let’s think step by step” to encourage the LLM to reason through the problem systematically [35]. While the prompting technique proved effective for other problems, it did not result in significant improvements for multilayer thin-film problems. The LLM frequently produced incorrect formulations when attempting to cascade multiple layers (see Supplementary materials Figure S5), highlighting the limitations of this simple method for addressing multilayer scenarios.

To address these challenges, we adopted one-shot CoT prompting by including a detailed process within the prompt. Specifically, a concise lecture note on TMM was provided, offering step-by-step explanations of the underlying physical theory and problem-solving methodology. This document addressed key concepts, focusing on the construction of matrices to analyze optical systems, including the representation of boundary transitions and propagation within layers in matrix form and the assembly of the overall transfer matrix to determine optical responses. The contents of the lecture note are available in the Supplementary materials. Importantly, the document was a general reference on TMM and was not specifically tailored for LLMs, with similar materials readily available in various academic courses on optics. Using this document, the LLM significantly improved its problem-solving capabilities, achieving over 70 % accuracy even for complex multilayer structures up to 10 layers. Compared to the zero-shot results, common errors were alleviated, particularly those related to the additional information provided in the lecture notes, such as incorrectly defining coefficients, reversing the order of matrix multiplication, and omitting denominator terms. However, some errors still occurred, such as applying incorrect phase terms during the calculations (see Supplementary materials Figure S6).

Lastly, we employed few-shot prompting techniques, supplementing the prompts with a more comprehensive lecture note that provided two specific TMM solutions (Figure 2c). This lecture note not only outlined the general problem-solving steps for TMM but also detailed the procedures for single-layer and triple-layer cases. As a result, the LLM further reduced errors and demonstrated improved generalization when addressing multilayer structures of varying complexity (see Supplementary materials Figure S9). These findings highlight the effectiveness of providing more information in the prompt, leading to more accurate outcomes from the LLM.

The benchmark results show that the performance of LLMs in calculating nanophotonic device properties is highly dependent on the provided prompts. While neither the user nor the LLM has working knowledge of TMM a priori, providing the LLM a lecture note with a few illustrative examples was enough to guide the LLM in creating an accurate numerical simulator and generate the correct answer. This trend of results remains consistent when a different prompt style is used by the user (e.g., a more natural-language-like style) as shown in Supplementary mateials Table S3. This highlights the model’s ability to understand domain-specific knowledge and generate code for numerical computation through in-context learning.

### 3.2 Conversational design of multilayer films

We now give the LLM a more difficult challenge of designing the multilayer films, where the goal is to find the refractive indices and thicknesses that are optimal to achieve a target spectral response. To demonstrate the design capabilities, we conducted experiments on optimization of optical multilayer devices by an interactive conversation between the user and the LLM. As a proof-of-concept, we designed a bandpass filter that passes light within the 500–600 nm range while blocking light in the rest of the visible spectrum. Again, we assumed that the user does not have expert knowledge in optics.

**Figure 4:**
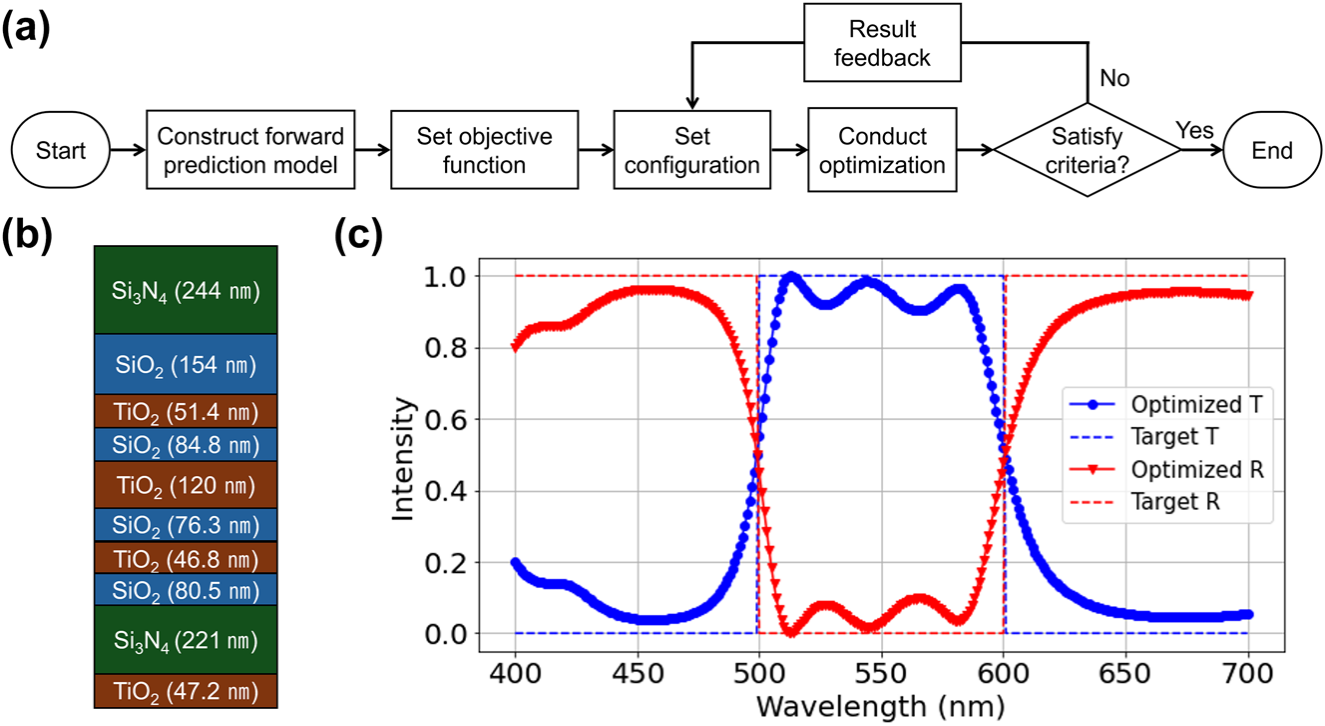
Inverse design of multilayer films through in-context learning. (a) Flowchart showing the conversational design workflow between a user and an LLM. (b) Configuration of the optimized multilayer film, consisting of 10 layers with a total thickness of 1,126 nm. (c) Spectral response of the optimized bandpass filter.

Figure 4a illustrates the flowchart of the conversational design process. Building on the forward model generated in the previous section, we ask LLM to define the objective function of the optimization problem. We incorporated prompts such as “I aim to develop optimization code for designing multilayer films to create a bandpass filter operating within the 500–600 nm range of the visible spectrum.” Subsequently, the model formulated objective function as the mean squared error (MSE) between the desired and calculated spectral values:

(1) $$f_{\mathrm{obj}}=\frac{1}{N}\overset{N}{\underset{i}{\sum }}{\left(T_{i}-{\hat{T}}_{i}\right)}^{2}$$

where *T*_*i*_ and ${\hat{T}}_{i}$ represent current and target transmission, at wavelength point *i*.

After defining the objective function through prompting, the initial structural configuration was established based on the constraints provided in the prompt and the generated material database. For instance, the design process began with settings such as a maximum number of layers of 5 and a total thickness of 2 μm. For material selection, the LLM recommended dielectric materials, including silicon dioxide (SiO_2_), silicon nitride (Si_3_N_4_), and titanium dioxide (TiO_2_). For the optimization, a gradient-free algorithm such as differential evolution was chosen for its effectiveness in exploring high-dimensional parameter spaces.

The design parameters and constraints were updated based on user feedback regarding the optimization results. The code is iteratively generated with adjusted material properties and structural configurations. Detailed prompts and code examples can be found in Supplementary materials. This approach resulted in an optimized 10-layer structure specifying the material and thickness of each layer, as shown in Figure 4b. The structure demonstrated an average transmittance exceeding 90 % within the specified wavelength range and dropping below 10 % outside that range, as illustrated in Figure 4c.

Our results indicate that interactive collaboration between users and AI allows design optimization without the need for in-depth knowledge of optical theory or numerical optimization. Through iterative feedback prompts, the code undergoes continuous refinement, progressively adapting to the specific requirements of the task. The final version of the code and its performance appear to closely match the quality typically achieved by domain experts.

## 4 Fine-tuning

For more complex nanophotonic systems, full-wave vectorial simulations are usually required and the corresponding codes are quite long and complicated, with several dedicated commercial products available. Generating these codes from scratch in a day is unlikely, even with the help of the current generation of LLMs. Moreover, these full-wave simulations may take several orders of magnitude longer to run compared to TMM simulations. For these reasons, alternative ways of tackling these problems using data-based methods seem attractive. In this section, we fine-tuned a pretrained LLM using a nanophotonic dataset for forward prediction and inverse design. As a specific example, we chose a wavelength filter made of periodic dielectric metasurfaces, which can modulate spectral properties based on their structural configurations [36], [37], [38].

### 4.1 Prediction of spectral responses of all-dielectric metasurfaces

**Figure 5:**
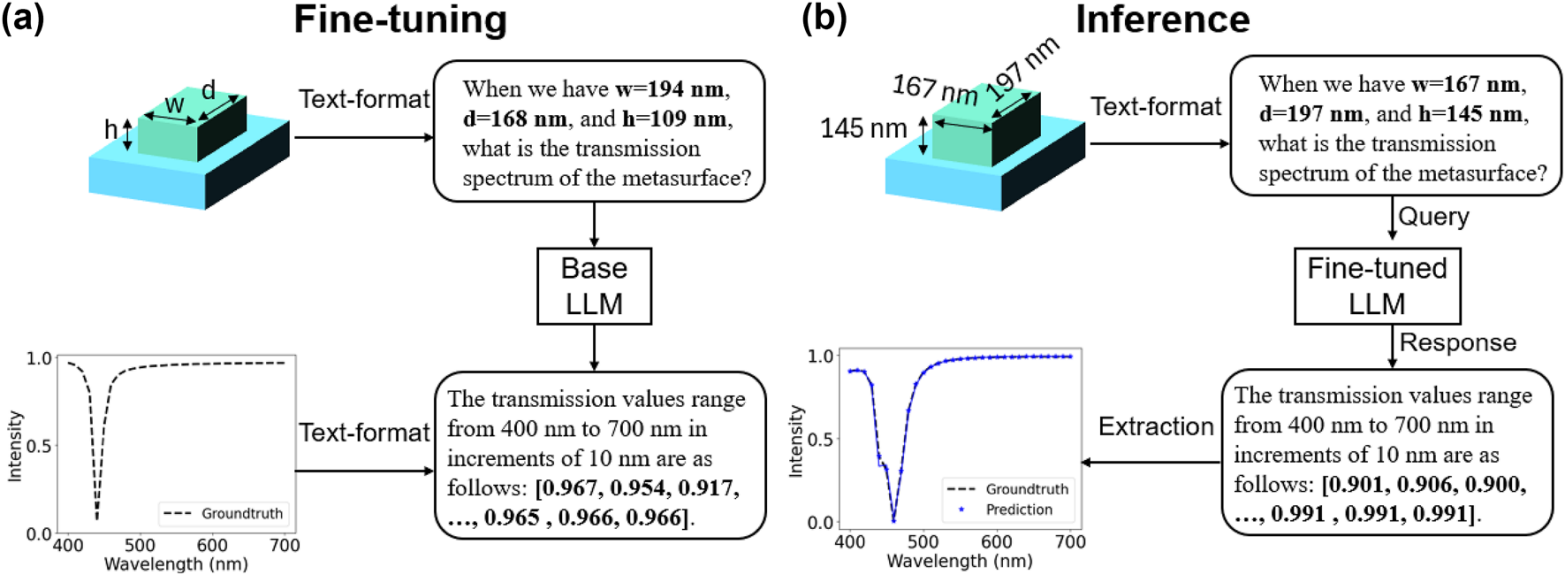
Language-interfaced fine-tuning for predicting the properties of optical metasurfaces. (a) Fine-tuning process. Both metasurfaces and spectral response data are transformed into text format. The base LLM adjusts its parameters to establish the relationship between geometry and transmittance. (b) Inference phase. The fine-tuned LLM predicts the spectrum when provided with new input data.

Figure 5a depicts the unit cell of the metasurface, consisting of a TiO_2_ cuboid on a SiO_2_ substrate. The metasurface has a period of 300 nm, with its cuboid scatterers characterized by three design parameters: width (*w*), depth (*d*), and height (*h*). The metasurface is illuminated by an x-polarized plane wave at normal incidence. Depending on the size parameters, the metasurface may have one or more resonances in the visible wavelength range and the transmission would be reduced around those resonant wavelengths.

We created a metasurface dataset using FDTD simulations with randomly chosen structural parameters as inputs. The spectral transmittance was recorded at 10 nm intervals between 400 nm and 700 nm after each simulation, resulting in an output vector with 31 points. The dataset comprises 2,000 training samples and 500 test samples.

To fine-tune the LLM for spectral transmittance prediction, we utilized the language-interfaced fine-tuning framework [27]. We converted a metasurface geometry into a textual format that the LLM can interpret directly. For example, a sample metasurface configuration is represented in a prompt as: “When we have *w* = 194 nm, *d* = 168 nm, and *h* = 109 nm, what is the transmission spectrum of the metasurface?” The corresponding transmission value vector is likewise represented in text format: “The transmission values range from 400 nm to 700 nm in increments of 10 nm are as follows: [0.967, 0.954, 0.917 …, 0.965, 0.966, 0.966].”

We utilized Meta’s Llama 3.1-8B-Instruct model [39] as the base LLM. Both the input and output texts were tokenized using the same tokenizer utilized during Llama 3 pretraining. Fine-tuning was conducted using LoRA on a single RTX 4090 GPU with the Unsloth framework. The rank *r* and the scaling factor *α* were set to 16 and 32, respectively, with a batch size of 20. The training process was carried out over 20 epochs using the AdamW optimizer with a learning rate of 3.00e-4. The fine-tuning employed the cross-entropy loss function used in the original language tasks for next-token prediction.

After fine-tuning the LLM, we evaluated its performance using a test set. Figure 5b represents the inference phase, which involves three key steps for language-based optical property prediction. First, the metasurface geometry is converted into a textual representation. Next, the fine-tuned LLM generates predictions by responding to the input text query. Finally, numerical data are extracted by parsing the generated text. As shown in the resulting graph, the predicted spectrum aligns closely with the ground truth values. We observed a mean squared error (MSE) of 7.23e-3 across the test samples, demonstrating the high accuracy of the model in capturing and predicting complex spectral features within the given structural parameter range (c.f. Supplementary materials for extrapolation). The performance of the model improves as the amount of training data increases, with the MSE decreasing and showing a trend toward saturation when the dataset size exceeds 2000 (see Supplementary materials Figure S1). Furthermore, we conducted experiments using other open-weights LLMs, and models with more than three billion parameters demonstrated comparable prediction accuracy, emphasizing the general applicability of LLM-based learning (see Supplementary materials Table S1).

As a side note, one out of the 500 inferences produced invalid outputs. They generated an inconsistent number of predicted values. This issue may arise from the probabilistic nature of generative models, which operate simply by predicting each successive word. However, in our experiments, regenerating the output consistently resolved such invalid results.

We also compared our approach with traditional neural network models. Specifically, we built a multilayer perceptron (MLP) with an input layer consisting of three design parameters and an output layer comprising 31 spectral points. The MLP demonstrated lower prediction error (MSE of 4.39e-3) and superior computational efficiency (<1 s). While traditional neural networks exhibit faster computation and higher accuracy in this case, they typically require task-specific architectural adjustments and lack the flexibility to handle a wide range of problems with a unified approach. We emphasize that the primary goal of fine-tuning LLMs is not to surpass traditional methods in accuracy or efficiency but to demonstrate the concept of “no-code machine learning” made possible by language-interfaced fine-tuning, as it eliminates the need for modifications to the underlying architecture, loss function, and hyperparameter optimization.

### 4.2 Inverse design of all-dielectric metasurfaces

**Figure 6:**
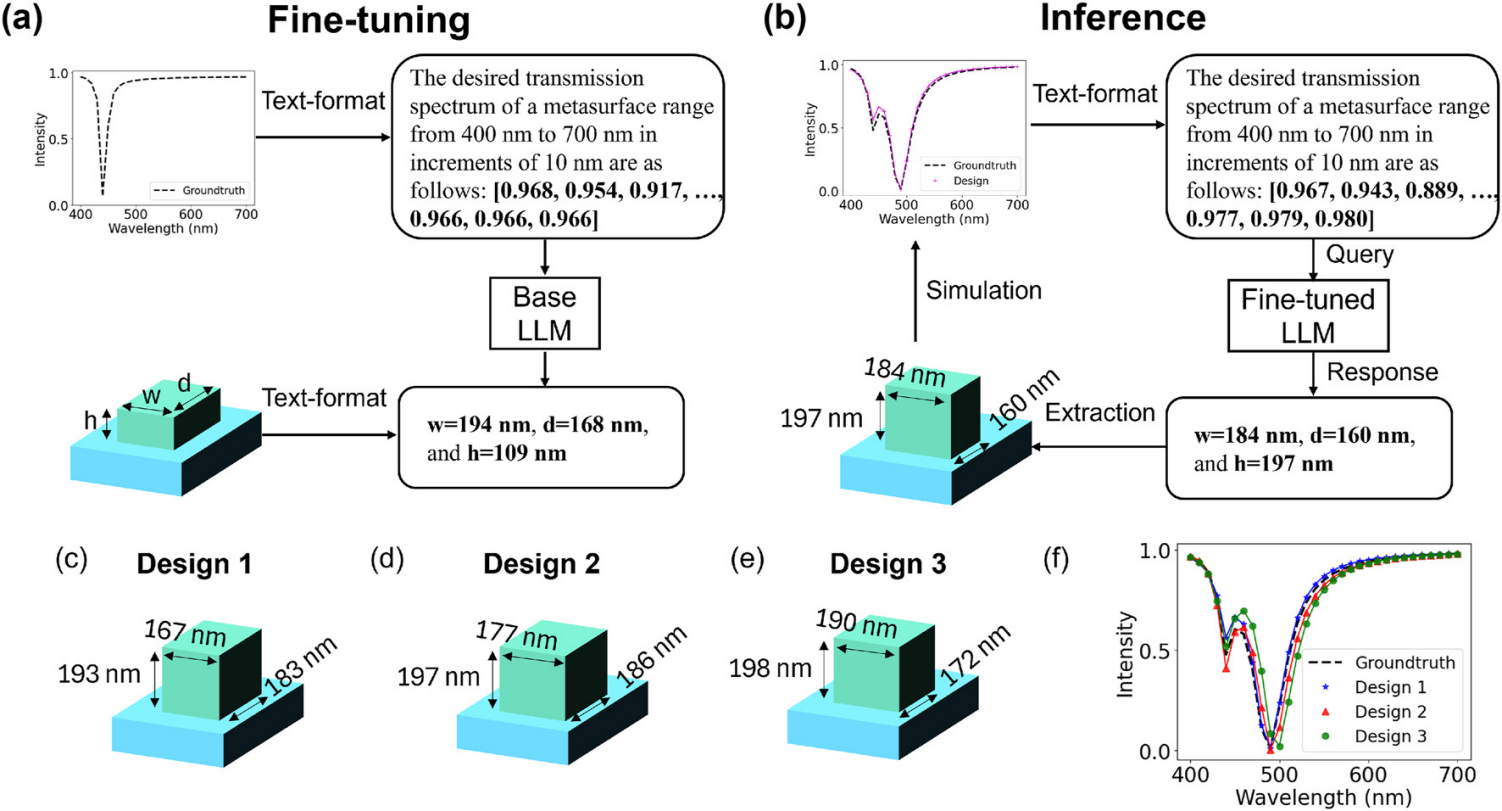
Language-interfaced fine-tuning for the inverse design of optical metasurfaces. (a) Workflow for fine-tuning process. The input text describes the desired properties, and the output provides the corresponding geometries. (b) Inference phase. The fine-tuned LLM takes new transmission as input and generates the corresponding metasurface structure. (c–e) Designs produced from independent runs, each with similar transmission but distinct parameter values. (f) The transmission of various structures generated by the LLM is compared against the target transmission.

To demonstrate the capabilities for design problems, we fine-tuned a base LLM to produce the unit cell structure of an all-dielectric metasurface that possesses the target spectral transmittance. The essential idea is to reverse the input–output relationship, with the spectrum provided as a string input and the structural parameters generated as textual output. As illustrated in Figure 6a, the input defines the desired optical properties, for example: “The desired transmission spectrum of a metasurface range from 400 nm to 700 nm in increments of 10 nm are as follows: [0.968, 0.954, 0.917,…, 0.966, 0.966, 0.966].” Similarly, the corresponding output specifies the geometric parameters, such as “w = 194 nm, d = 168 nm, and h = 109 nm.” After fine-tuning the base LLM with the reversed dataset, the LLM produces text-based descriptions of the structural parameters required to achieve the target spectral response, which are subsequently used to determine specific configurations. The resulting structure is then simulated, and its spectral response is compared with the input target spectrum (Figure 6b).

A notable feature of LLMs is their ability to produce diverse outputs even for the same target objective. This flexibility is particularly useful in inverse design tasks, as it enables the discovery of new design possibilities and expands the range of potential solutions. Figure 6c–e showcases the results of multiple tests, highlighting the variety of solutions generated by the model.

The diversity in outputs can be primarily controlled and refined by adjusting two key hyperparameters: temperature and the top-p sampling threshold, which shape the probability distribution of the generated output tokens. The temperature governs the randomness of the responses to the model. Formally, the probability of a token being selected as the next token is defined as:

(2) $$p_{i}=\frac{\exp\left(z_{i}/\tau \right)}{\underset{j}{\sum }\exp\left(z_{j}/\tau \right)}$$

where *z*_*i*_ is the logit of token, *p*_*i*_ represents its selection probability, and *τ* is the temperature. Intuitively, increasing the temperature leads to more diverse and creative outputs, while decreasing it results in more deterministic generations. Top-p sampling, on the other hand, selects tokens from the smallest set whose cumulative probability exceeds a threshold *p*, ensuring that the generation focuses on the most likely tokens. We set the temperature to *τ* = 2.0 and *p* = 0.9 for the experiments. Metasurfaces were generated across four independent runs, with all resulting structures having distinct designs yet demonstrating similar spectral responses, as depicted in Figure 6b and f.

Our findings suggest that language-interfaced fine-tuning of LLMs is effective for both forward prediction and inverse design of metasurfaces. In both cases, only the number-to-text encoding and text-to-number decoding processes are required, with the core fine-tuning process remaining consistent and not demanding in-depth knowledge of AI models. Moreover, the model exhibits flexibility in handling diverse input representations, such as resonance wavelength and bandwidth (see Supplementary materials Figure S3). Consequently, this method lowers the barrier for researchers with limited expertise in AI or physics, enabling them to participate in data-driven nanophotonic design tasks with ease and efficiency.

## 5 Conclusions

In conclusion, we have evaluated two distinct capabilities of large language models for addressing nanophotonic design problems. Using few-shot chain-of-thought prompting, the transmission and reflection of multilayer devices can be accurately predicted, even without prior knowledge of multilayer films from either the researcher or the model if a domain-specific, “lecture note” text is available. Additionally, we demonstrated that the in-context learning capability of LLMs can be extended to the inverse design process through iterative, conversational prompting with researchers, producing optimal multilayer film designs for given optical specifications. Through fine-tuning with a text-formatted dataset, we achieved accurate spectral response predictions for queried structures, while also identifying metasurface designs that met the desired spectral characteristics. Importantly, our approach is not limited to a specific LLM; therefore, we anticipate enhanced design capabilities by substituting it with more advanced models.

We outline two potential directions for future research. One approach involves integrating multimodal LLMs into the design process, potentially broadening the range of design problems. For example, Llama-3.2 accepts both images and text as input, allowing forward prediction and inverse design of freeform structures by representing the structure as a pixelated array and spectral data as text. In addition, incorporation of tunable materials such as phase-change materials could be considered. Another promising direction is to develop AI agents capable of autonomously gathering data, making design decisions, and interacting with users, thereby accelerating the development of innovative nanophotonic devices.

## Supplementary Material

Supplementary Material Details
