# Supplementary material for: Nanophotonic device design based on large language models: multilayer and metasurface examples
Source: Nanophotonics. 2025 Feb 17;14(8):1273–82. doi: 10.1515/nanoph-2024-0674 (PMC12019936; doi:10.1515/nanoph-2024-0674)
Supplement: Supplementary file 1 — Supplementary Material Details [file j_nanoph-2024-0674_suppl_001.pdf]

Supplementary Materials

Nanophotonic device design based on large language models: multilayer and metasurface examples

Myungjoon Kim, Hyeonjin Park, and Jonghwa Shin
Department of Materials Science and Engineering
KAIST, Daejeon, Republic of Korea

I. COMPARISON WITH OTHER LLMS

In the main text, Llama-3.1-8B is mainly used for fine-tuning process. In this section, we additionally tested whether other LLMs can have similar performance. We conducted a benchmark test using three additional LLMs: Llama-3.2-1B, Llama-3.2-3B, and Mistral-7B. The mean squared error (MSE) and the number of invalid outputs for each model are presented in Table S1. The results indicate that models with more than 3 billion parameters demonstrated comparable prediction accuracy, emphasizing the general applicability of LLM-based learning.

TABLE S1. Learning performance benchmark results across different LLMs.

Model	MSE	No. of invalid outputs
Llama-3.2-1B	1.70e-2	8
Llama-3.2-3B	8.25e-3	2
Llama-3.1-8B	7.23e-3	1
Mistral-7B	8.35e-4	0

II. IMPACT OF DATA SIZE ON PREDICTION ACCURACY

The key advantage of pre-trained LLMs lies in their ability to adapt efficiently to new downstream tasks with relatively small datasets. Using 2000 samples demonstrates this efficiency, reflecting the practical scale of photonic simulations, where such a dataset size is achievable within a reasonable computational timeframe.

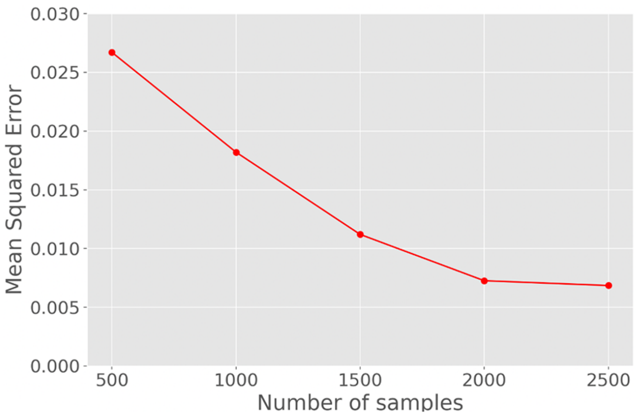

FIG. S1. Impact of data quantity on model performance.

To investigate further the effect of data quantity on model performance, we conducted a benchmark with different numbers of training examples (500, 1000, 1500, 2000, and 2500). As illustrated in Figure S1, the prediction error decreases as the number of samples increases. Notably, performance begins to saturate beyond 2000 samples, offering a balance between practicality and effectiveness.

III. ANALYSIS OF INTERPOLATION AND EXTRAPOLATION PERFORMANCE

In the main text, the fine-tuned LLM model is tested solely on "interpolation," meaning the range of variables in the test dataset is the same as that in the training dataset. To explore the model's capability for extrapolation, we conducted an additional experiment with new test dataset.

We first define the extrapolation data as containing at least one variable that fall outside the range of the training set. To quantify this, we first define the *distance* to the training dataset for each structure. This distance is measured as the Euclidean distance between a given structure and its closest neighbor in the training set. Mathematically, the distance L is defined as

$$L(w, d, h) = \min_{(w_i, d_i, h_i) \in \mathcal{X}_{train}} \sqrt{(w - w_i)^2 + (d - d_i)^2 + (h - h_i)^2}$$

where w, d, and h is the width, depth, and height of the meta-atom geometry, and $\mathcal{X}_{train}$ denotes the set of training input vectors. Figure S2 presents a scatter plot showing the relationship between distance and error, highlighting that the extrapolation dataset frequently exhibits higher error rates.

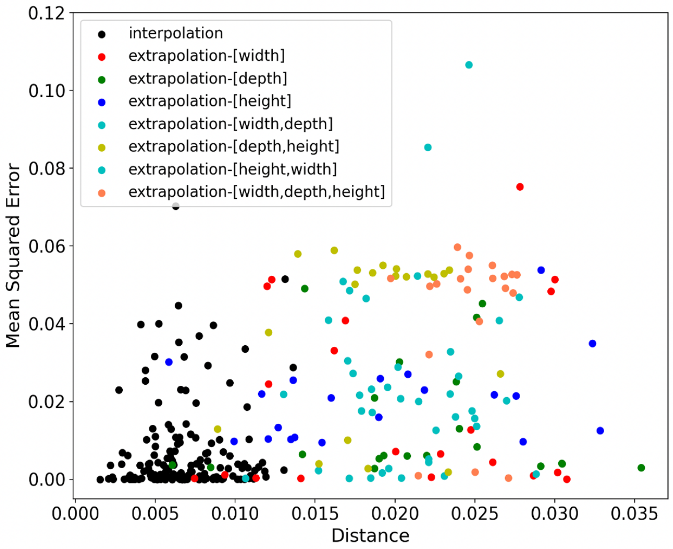

FIG. S2. Prediction error for interpolation and extrapolation data. Each color represents a different data type. For example, *extrapolation-[height,width]* indicates structures with height and width outside the range of the training dataset.

IV. FINE-TUNING WITH ALTERNATIVE INPUT REPRESENTATION

The spectral responses of periodic metasurfaces can be expressed in alternative formats, such as resonance wavelengths and bandwidths. To evaluate whether the fine-tuned LLM can effectively handle this type of input representation, we used the same dataset but first filtered out structures with non-resonant characteristics, reducing the total from 2000 to 1708. For the inverse design task, the input was specified as: "The desired wavelength and bandwidth are 470 nm and 50 nm." Figure S3 demonstrates the model's ability to adapt to this format, highlighting its versatility in managing various representations for inverse design applications.

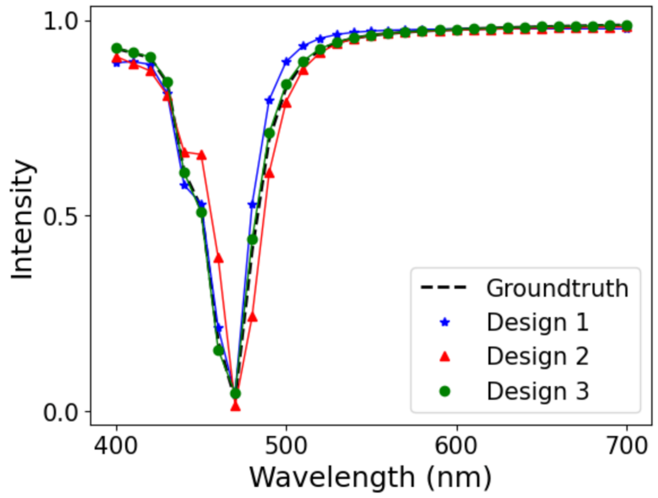

FIG. S3. Inverse design results with alternative input representations.

V. STASTICAL ANALYSIS OF IN-CONTEX LEARNING BENCHMARK RESULTS

Table S2 summarizes the experimental results of ICL with various prompt techniques in the form of 'number of successes / total attempts'.

TABLE S2. Benchmark results for numerical simulation of multilayer films using code generated by ChatGPT. We report the number of correct responses obtained across 50 trials for each of the four different prompt engineering techniques. Experiment conducted on January 15, 2025.

	1 Layer	2 Layers	3 Layers	5 Layers	10 Layers
Zero-shot	6/50	8/50	7/50	8/50	5/50
Zero-shot CoT	7/50	8/50	8/50	5/50	2/50
One-shot CoT	46/50	45/50	38/50	38/50	35/50
Few-shot CoT	50/50	48/50	42/50	42/50	44/50

In this study, because the LLM's output tends either to precisely match the groundtruth or deviate significantly, conventional error measures such as MSE are less meaningful. Instead, we treated the success rate in Table S2 as a binomial distribution and applied a 95% confidence interval for the success rate using the Wilson interval calculation formula. Specifically, for each success count k out of n total attempts, we calculate the adjusted success

rate $\hat{p}$, the adjusted standard error SE, and finally the confidence interval CI according to the formula:

$$\hat{p} = \frac{k + z^2/2}{n + z^2}, \quad SE = \sqrt{\frac{\hat{p}(1-\hat{p})}{n + z^2}}, \quad CI = \hat{p} \pm z \cdot SE,$$

where z is the z-score corresponding to the desired confidence level (e.g., $z = 1.96$ for a 95% confidence interval). This approach provides a more robust estimate of uncertainty in scenarios where the model is either completely accurate or exhibits substantial deviations. Figure 3 in the manuscript illustrates the adjusted success rate and its corresponding confidence interval for each problem case.

VI. PROMPT EXAMPLES

In this work, we employed four different prompting techniques: zero-shot, zero-shot CoT, one-shot CoT, and few-shot CoT. Examples of prompts and their corresponding responses for each technique are presented in Figures S4–S9.

VII. EXAMPLE OF TRANSFER MATRIX METHOD DOCUMENTATION

In the in-context learning approach, we used a prompt featuring descriptions of the transfer matrix method (TMM) to assist the LLM in predicting the spectral response of multilayered structures. The prompt included a general explanation of the TMM and two example problems: one for a single-layer structure and the other for a triple-layer structure. The complete details are provided in the document below.

A. Description

The transfer matrix method (TMM) is used to determine the reflection and transmission properties of a multilayered structure. In TMM, each layer is represented by a characteristic matrix C_i, which relates the electromagnetic fields on the reflection side to those on the transmission side of the ith layer. For a structure with N layers, the overall transfer matrix M is formed by cascading these N characteristic matrices, as shown below:

$$M = \prod_{i=1}^{N} C_i = C_1 C_2 C_3 \ldots C_{N-1} C_N$$

The characteristic matrix C_i for the ith layer is composed of two specific matrices: the propagation matrix P_i, which accounts for the propagation phase within the ith layer, and the transition matrix $T_{(i-1,i)}$, which describes the material transition at the boundary between the $(i-1)^{\text{th}}$ and the ith layers. The propagation and the transition matrices are defined as follows, where n_i represents the refractive index, t_i denotes the thickness, and η_i is the wave impedance, which is inversely proportional to the refractive index, for the ith layer.

$$P_i = \begin{bmatrix} \exp(-in_i k_0 t_i) & 0 \\ 0 & \exp(in_i k_0 t_i) \end{bmatrix}$$

$$T_{(i-1,i)} = \frac{1}{2} \begin{bmatrix} 1 + \frac{\eta_{i-1}}{\eta_i} & 1 - \frac{\eta_{i-1}}{\eta_i} \\ 1 - \frac{\eta_{i-1}}{\eta_i} & 1 + \frac{\eta_{i-1}}{\eta_i} \end{bmatrix}$$

The transfer matrix M then can be expressed in terms of the propagation and the transition matrices as follows:

$$M = \prod_{i=1}^{N} C_i = C_1 C_2 \ldots C_{N-1} C_N = T_{(0,1)} P_1 T_{(1,2)} P_2 \ldots T_{(N-2,N-1)} P_{N-1} T_{(N-1,N)} P_N$$

When an incident field passes through the multilayered structure from air, we also account for the transition between the last N^{th} layer and the transmission-side air, denoted by T_{last}. By multiplying this last transition matrix $T_{last} = T_{(N,air)}$ with the transfer matrix M, we obtain the final transfer matrix M_{final} as shown below:

$$M_{final} = M T_{last} = T_{(0,1)} P_1 T_{(1,2)} P_2 \ldots T_{(N-2,N-1)} P_{N-1} T_{(N-1,N)} P_N T_{(N,air)}$$

From the matrix M_{final}, the reflection coefficient r and the transmission coefficients t can be determined as follows:

$$t = \frac{1}{M_{final}(1,1)}$$

$$r = \frac{M_{final}(2,1)}{M_{final}(1,1)}$$

Finally, the reflectance R and the transmittance T are obtained by taking the absolute squares of the coefficients:

$$R = |r|^2$$

$$T = |t|^2$$

B. Example 1

Question: When a plane wave with a wavelength of 400 nm is incident perpendicularly, what are the reflectance and transmittance of the following structure?

Structure: Air/Slab 1/Air
- Slab1: thickness 50 nm, refractive index $1.2 + 1i \times 0.01$
Process: First, the material properties are defined as follows:

$$Air : n_{air} = n_0 = 1, \eta_{air} = \frac{\eta_0}{n_{air}} = \eta_0, t_{air} = 0$$

$$Slab1 : n_1 = 1.2 + 1i \times 0.01, \eta_1 = \frac{\eta_0}{n_1}, t_1 = 50 \times 10^{-9}$$

The problem involves a single slab ($N = 1$) surrounded by air. Therefore, the final transfer matrix is calculated as:

$$M_{final} = MT_{last} = \prod_{i=1}^{1} C_i T_{last} = C_1 T_{last} = T_{0,1} P_1 T_{1,air}$$

The corresponding matrices are defined as follows:

$$T_{0,1} = \frac{1}{2} \begin{bmatrix} 1 + \frac{\eta_0}{\eta_1} & 1 - \frac{\eta_0}{\eta_1} \\ 1 - \frac{\eta_0}{\eta_1} & 1 + \frac{\eta_0}{\eta_1} \end{bmatrix}$$

$$T_{last} = T_{1,air} = \frac{1}{2} \begin{bmatrix} 1 + \frac{\eta_1}{\eta_0} & 1 - \frac{\eta_1}{\eta_0} \\ 1 - \frac{\eta_1}{\eta_0} & 1 + \frac{\eta_1}{\eta_0} \end{bmatrix}$$

$$P_1 = \begin{bmatrix} \exp(-in_1 k_0 t_1) & 0 \\ 0 & \exp(in_1 k_0 t_1) \end{bmatrix}$$

The final transfer matrix is then computed as:

$$M_{final} = C_1 T_{last} = T_{0,1} P_1 T_{1,air}$$

From the components of this matrix, the reflection (r) and transmission (t) coefficients are determined as:

$$t = \frac{1}{M_{final}(1,1)}$$

$$r = \frac{M_{final}(2,1)}{M_{final}(1,1)}$$

The reflectance (R) and transmittance (T) are then calculated as:

$$R = |r|^2$$

$$T = |t|^2$$

Using these equations, the results are obtained as:

$$R = 0.021$$

$$T = 0.962$$

Answer: The reflectance (R) and the transmittance (T) is $R = 0.021$, T=0.962

C. Example 2

Question: When a plane wave with a wavelength of 630 nm is incident perpendicularly, what are the reflectance and transmittance of the following structure in the air?

Structure: Air/Slab 1/Slab 2/Slab 3/Air
- Slab1: thickness 50 nm, refractive index $1.3 + 1i \times 0.02$
- Slab2: thickness 20 nm, refractive index $0.3 + 1i \times 1.4$
- Slab3: thickness 40 nm, refractive index $1.8 + 1i \times 0.01$

Process: First, the material properties are defined as follows:

$$Air : n_{air} = n_0 = 1, \eta_{air} = \frac{\eta_0}{n_{air}} = \eta_0, t_{air} = 0$$

$$Slab1 : n_1 = 1.3 + 1i \times 0.02, \eta_1 = \frac{\eta_0}{n_1}, t_1 = 50 \times 10^{-9}$$

$$Slab2 : n_2 = 0.3 + 1i \times 1.4, \eta_2 = \frac{\eta_0}{n_2}, t_2 = 20 \times 10^{-9}$$

$$Slab3 : n_3 = 1.8 + 1i \times 0.01, \eta_3 = \frac{\eta_0}{n_3}, t_3 = 40 \times 10^{-9}$$

The problem involves a triple slab ($N = 3$) surrounded by air. Therefore, the final transfer matrix is calculated as:

$$M_{final} = MT_{last} = \prod_{i=1}^{3} C_i T_{last} = C_1 C_2 C_3 T_{last} = T_{0,1} P_1 T_{1,2} P_2 T_{2,3} P_3 T_{3,air}$$

The corresponding matrices are defined as follows:

$$T_{0,1} = \frac{1}{2}\begin{bmatrix} 1+\frac{\eta_0}{\eta_1} & 1-\frac{\eta_0}{\eta_1} \\ 1-\frac{\eta_0}{\eta_1} & 1+\frac{\eta_0}{\eta_1} \end{bmatrix}$$

$$T_{1,2} = \frac{1}{2}\begin{bmatrix} 1+\frac{\eta_1}{\eta_2} & 1-\frac{\eta_1}{\eta_2} \\ 1-\frac{\eta_1}{\eta_2} & 1+\frac{\eta_1}{\eta_2} \end{bmatrix}$$

$$T_{2,3} = \frac{1}{2}\begin{bmatrix} 1+\frac{\eta_2}{\eta_3} & 1-\frac{\eta_2}{\eta_3} \\ 1-\frac{\eta_2}{\eta_3} & 1+\frac{\eta_2}{\eta_3} \end{bmatrix}$$

$$T_{last} = T_{3,air} = \frac{1}{2}\begin{bmatrix} 1+\frac{\eta_3}{\eta_0} & 1-\frac{\eta_3}{\eta_0} \\ 1-\frac{\eta_3}{\eta_0} & 1+\frac{\eta_3}{\eta_0} \end{bmatrix}$$

$$P_1 = \begin{bmatrix} \exp(-in_1k_0t_1) & 0 \\ 0 & \exp(in_1k_0t_1) \end{bmatrix}$$

$$P_2 = \begin{bmatrix} \exp(-in_2k_0t_2) & 0 \\ 0 & \exp(in_2k_0t_2) \end{bmatrix}$$

$$P_3 = \begin{bmatrix} \exp(-in_3k_0t_3) & 0 \\ 0 & \exp(in_3k_0t_3) \end{bmatrix}$$

The final transfer matrix is then computed as:

$$M_{final} = C_1C_2C_3T_{last} = T_{0,1}P_1T_{1,2}P_2T_{2,3}P_3T_{3,air}$$

From the components of this matrix, the reflection (r) and transmission (t) coefficients are determined as:

$$t = \frac{1}{M_{final}(1,1)}$$

$$r = \frac{M_{final}(2,1)}{M_{final}(1,1)}$$

The reflectance (R) and transmittance (T) are then calculated as:

$$R = |r|^2$$

$$T = |t|^2$$

Using these equations, the results are obtained as:

$$R = 0.066$$

$$T = 0.823$$

Answer: The reflectance (R) and the transmittance (T) is $R = 0.066, T = 0.823$

VIII. USER DEPENDENT PROMPTING STYLE

The prompt components beyond the lecture notes (such as problem definitions and structural descriptions) can vary depending on the user's approach. To verify the user-independent effectiveness of the ICL approach, we conducted additional experiments using the same lecture materials but with varying forms of problem-setting prompts. While the original prompt included ### and used well-organized format, the additional experiments employed unstructured, prose-style prompts, as illustrated in the following example. We conducted 30 experiments for each case and the result summarized in Table S3.

> "I want to calculate the transmittance and reflectance for a triple slab at a wavelength of 400 nm.
> The refractive index of Slab 1 is 1.45 with a thickness of 50 nm, the refractive index of Slab 2 is 0.64 + 2.98i with a thickness of 25 nm, and the refractive index of Slab 3 is 1.768 with a thickness of 50 nm.
> What are the values?"

TABLE S3. Benchmark results for different prompt style. Experiment conducted on January 09, 2025.

	1 Layer	2 Layers	3 Layers	5 Layers	10 Layers
Zero-shot	1/30	7/30	8/30	3/30	3/30
Zero-shot CoT	1/30	2/30	6/30	1/30	3/30
One-shot CoT	27/30	29/30	26/30	25/30	25/30
Few-shot CoT	29/30	30/30	27/30	28/30	25/30

The result demonstrates a good alignment with the original experiments. This consistency suggests that ICL effectively enhances the accuracy of LLM responses, even with varying prompt styles. Based on these observations, we believe that ICL is broadly applicable, regardless of differences in user input style.

IX. PROMPT FOR CONVERSATIONAL DESIGN

For the conversational design of multilayer thin films, we prompted the LLM according to the workflow outlined in Figure 4a in the main text. We included the specific prompts used for each step, along with the corresponding code generated by ChatGPT in response.

A. Construct forward prediction model

We provided the document "Supplementary Materials VII.A–VII.C" and prompted the LLM with, "Using the information, develop a code that can be generally applied to retrieve the reflectance and transmittance of an N-layered structure across multiple wavelengths."

In response, the LLM generated forward prediction code as shown in Figure S10

B. Set objective function

We prompted the LLM with: “I aim to develop optimization code for designing multilayer films to create a bandpass filter operating within the 500–600 nm range of the visible spectrum. Generate code for the objective function.”

The LLM generated an appropriate objective function, such as the mean square error between the desired and designed responses, as shown in Figure S11.

C. Set configuration

We prompted the LLM with: “Let’s consider a 5-layer structure and each layer is a realistic dielectric material that can be used in actual fabrication. The upper limit of the total thickness is 2 microns. Generate code for the configuration.”

The LLM generated configurational parameters and constraints, including maximum thickness, number of layers, and realistic material property data, as shown in Figure S12

D. Conduct optimization

We prompted the LLM with: “Generate code for the entire optimization process with a plot for the final result."

The LLM provided the full optimization code, incorporating the appropriate optimization algorithm such as the evolutionary algorithm (Figure S13). We then executed the optimization and attained the result shown in Fig S14.

E. Result feedback

We prompted the LLM with: “The optimization results are unsatisfactory. Let’s increase the number of layers and try a different configuration. Please regenerate the optimization code."

In response, the LLM regenerated code with the NUM_LAYERS parameter updated, and offered additional material data such as Si_3N_4, and Hf_2O. Figure S15 shows the result from the modified code in the feedback process. The result shown in Figure 4c in the main text is obtained from the modified code in the final conversational loop (Figure S16).

(a) User

Question: When a plane wave with a wavelength of 400 nm is incident perpendicularly, what are the reflectance and transmittance of the following structure?

Structure: Air/Slab 1/Air
- Slab 1: thickness 30 nm, refractive index 2.1 + 1i*0.03

(b) LLM

1. **Reflection and Transmission at an Interface:** The reflection coefficient r and transmission coefficient t at the interface between two media with refractive indices n_0 and n_1 are given by:

$$r = \frac{n_0 - n_1}{n_0 + n_1}$$

$$t = \frac{2n_0}{n_0 + n_1}$$

2. **Phase Accumulation in the Slab:** As the wave propagates through the slab, it accumulates a phase ϕ given by:

$$\phi = \frac{2\pi n_1 d_1}{\lambda}$$

where n_1 is the complex refractive index of the slab, d_1 is the thickness of the slab, and λ is the wavelength in vacuum.

3. **Overall Reflection and Transmittance:** For a single thin slab, the total reflectance R and transmittance T are related to the coefficients r and t, and the phase shift inside the slab, with:

$$R = |r_{total}|^2$$

$$T = \frac{|t_{total}|^2 n_1}{n_0}$$

(c) LLM

```
import numpy as np

# Given parameters
n_air = 1  # Refractive index of air
n_slab = 2.1 + 1j*0.03  # Refractive index of slab 1 (complex)
thickness_slab = 30e-9  # Thickness of slab 1 in meters (30 nm)
wavelength = 400e-9  # Wavelength in meters (400 nm)

# Reflection and transmission coefficients at air-slab interface
r_01 = (n_air - n_slab) / (n_air + n_slab)
t_01 = (2 * n_air) / (n_air + n_slab)

# Phase accumulation in the slab
phi = (2 * np.pi * n_slab * thickness_slab) / wavelength

# Overall reflectance and transmittance for single interface (no multiple reflections)
R = np.abs(r_01)**2
T = (np.abs(t_01)**2) * (np.real(n_slab) / n_air)

R, T
```

(d) LLM

The reflectance (R) and transmittance (T) of the given structure at a wavelength of 400 nm are:
- **Reflectance (R)**: 0.126 (or 12.6%)
- **Transmittance (T)**: 0.874 (or 87.4%)

These values indicate that approximately 12.6% of the incident light is reflected, while 87.4% is transmitted through the slab.

FIG. S4. Results of failed zero-shot prompting for a single-layer structure. (a) The user's input prompt. (b) The LLM explains the solution for the problem but makes an error in considering the interfaces. (c) The code generated based on the reasoning shows inconsistencies, including errors such as using only the real part of the refractive index to compute transmittance. (d) The LLM outputs incorrect final values for transmission and reflectance. The groundtruth values are $R = 0.3056$ and $T = 0.6631$.

(a)

User

Question: When a plane wave with a wavelength of 400 nm is incident perpendicularly, what are the reflectance and transmittance of the following structure?

Structure: Air/Slab 1/Slab 2/Slab 3/Air
- Slab 1: thickness 50 nm, refractive index 1.45
- Slab 2: thickness 25 nm, refractive index 0.64 + 1i * 2.98
- Slab 3: thickness 50 nm, refractive index 1.768

Let's think step by step.

(b)

LLM

Let's solve this step by step.
Step 1: Understanding the problem
We have a multilayer structure consisting of three slabs with the following properties:
1.Slab 1:
1. Thickness d_1=50 nm
2. Refractive index n_1=1.45

2. Slab 2:
1. Thickness d_2=25 nm
2. Refractive index n_2=0.64+1i·2.98 (complex refractive index, indicating absorption)

3.Slab 3:
1. Thickness d_3=50 nm
2. Refractive index n_3=1.768

The wavelength of the incident plane wave is λ_0=400 nm.
The incident medium and the final medium are air, with refractive index n_0=n_4=1 (for air).
We need to calculate the **reflectance** and **transmittance** of the structure when the plane wave is incident perpendicularly.

Step 2: Apply the Transfer Matrix Method
For a multilayer structure, the transfer matrix method is typically used to calculate the reflectance and transmittance. Each layer contributes a transfer matrix that depends on its refractive index and thickness. The total transfer matrix is the product of the individual matrices.
General form of the transfer matrix for a layer:
For a slab of refractive index n and thickness d, the transfer matrix is given by:

$$\begin{pmatrix} \cos(\delta) & \frac{isin(\delta)}{Z} \\ iZsin(\delta) & cos(\delta) \end{pmatrix}$$

where:
•$Z = \frac{n}{n_0}$ is the normalized impedance (with respect to air),
•$\delta = \frac{2\pi nd}{\lambda 0}$ is the phase thickness of the layer, where n is the refractive index and d is the thickness of the layer,
•λ_0 is the wavelength of the incident light in a vacuum.
Transfer matrix for the entire structure:
For a multilayer structure like this one (Air/Slab 1/Slab 2/Slab 3/Air), the total transfer matrix M_{total} is the product of the individual transfer matrices:
$M_{total} = M_1 \cdot M_2 \cdot M_3$ where $M_1, M_2, and\ M_3$ are the transfer matrices for the respective slabs.

Step 3: Calculate Reflectance and Transmittance
Once we have the total transfer matrix, the reflectance R and transmittance T can be calculated using:

$$R = \left|\frac{M_{total,1,1} + M_{total,1,2}Z_4 - Z_0\left(M_{total,2,1} + M_{total,2,2}Z_4\right)}{M_{total,1,1} + M_{total,1,2}Z_4 + Z_0\left(M_{total,2,1} + M_{total,2,2}Z_4\right)}\right|^2$$

$$T = \frac{4Z_0Z_4}{\left|M_{total,1,1} + M_{total,1,2}Z_4 + Z_0\left(M_{total,2,1} + M_{total,2,2}Z_4\right)\right|^2}$$

Where:
•$Z_0 = 1$ is the impedance of air (incident medium),
•$Z_4 = 1$ is the impedance of air (final medium).

Step 4: Implementing the Calculations
Let me calculate the necessary phase shifts δ for each slab and then determine the reflectance and transmittance using the transfer matrix method.
I'll compute these results for you now.

(c)

LLM

```
import numpy as np

# Given parameters
lambda_0 = 400  # wavelength in nm
n_air = 1.0  # refractive index of air

# Slab properties: thickness (nm) and refractive index (complex for slab 2)
d1, n1 = 50, 1.45
d2, n2 = 25, 0.64 + 1j*2.98
d3, n3 = 50, 1.768

# Impedances (normalized refractive index with respect to air)
Z1 = n1 / n_air
Z2 = n2 / n_air
Z3 = n3 / n_air

# Calculate phase thickness (delta) for each slab
delta1 = 2 * np.pi * n1 * d1 / lambda_0
delta2 = 2 * np.pi * n2 * d2 / lambda_0
delta3 = 2 * np.pi * n3 * d3 / lambda_0

# Transfer matrix for each slab
def transfer_matrix(delta, Z):
    return np.array([[np.cos(delta), 1j * np.sin(delta) / Z],
                     [1j * Z * np.sin(delta), np.cos(delta)]])

# Transfer matrices for the slabs
M1 = transfer_matrix(delta1, Z1)
M2 = transfer_matrix(delta2, Z2)
M3 = transfer_matrix(delta3, Z3)

# Total transfer matrix (product of individual matrices)
M_total = M1 @ M2 @ M3

# Impedance of air (initial and final medium)
Z0 = n_air  # air
Z4 = n_air  # air

# Reflectance and transmittance formulas
numerator_R = M_total[0, 0] + M_total[0, 1] * Z4 - Z0 * (M_total[1, 0] + M_total[1, 1] * Z4)
denominator_R = M_total[0, 0] + M_total[0, 1] * Z4 + Z0 * (M_total[1, 0] + M_total[1, 1] * Z4)
R = np.abs(numerator_R / denominator_R) ** 2

T = (4 * Z0 * Z4) / np.abs(M_total[0, 0] + M_total[0, 1] * Z4 + Z0 * (M_total[1, 0] + M_total[1, 1] * Z4))
** 2

R, T
```

(d)

LLM

The calculated **reflectance** of the structure is approximately 0.894 (or 89.4%), and the **transmittance** is approximately 0.728 (or 72.8%). This means a significant portion of the incident light is reflected, while some of it passes through the structure. The results take into account the complex refractive index of the second slab, which includes absorption.

FIG. S5. Results of failed zero-shot CoT prompting for a triple-layer structure. (a) The user's input prompt. (b) The LLM generates reasoning steps that are relevant but contain incorrect mathematical definitions, such as errors in the transfer matrix and normalized impedance. (c) The Python code produced based on the reasoning. (d) The output generated from executing the code. The correct groundtruth values are $R = 0.4421$ and $T = 0.2801$.

(a)

User

Provide documentation on the transfer matrix method (TMM).
1. General description of TMM

Question: When a plane wave with a wavelength of 400 nm is incident perpendicularly, what are the reflectance and transmittance of the following structure?

Structure: Air/Slab 1/Slab 2/Slab 3/Slab 4/ Slab 5/Air
- Slab 1: thickness 20 nm, refractive index 1.22
- Slab 2: thickness 25 nm, refractive index 2.1 + 1i*0.08
- Slab 3: thickness 30 nm, refractive index 0.54 + 1i*1.82
- Slab 4: thickness 20 nm, refractive index 1.34 + 1i*0.1
- Slab 5: thickness 40 nm, refractive index 2.48

(b)

LLM

To calculate the reflectance R and transmittance T for the given multilayer structure using the transfer matrix method (TMM), we will follow the steps outlined in your explanation. Here's a step-by-step breakdown of the process:

Given Parameters:

1.Wavelength $\boldsymbol{\lambda = 400\,nm}$ (incident wave).

2.Slabs with thicknesses and complex refractive indices as provided:

  1. Slab 1: $t_1 = 20\,nm$, $n_1 = 1.22$
  2. Slab 2: $t_2 = 25\,nm$, $n_2 = 2.1 + i0.08$
  3. Slab 3: $t_3 = 30\,nm$, $n_3 = 0.54 + i1.82$
  4. Slab 4: $t_4 = 20\,nm$, $n_4 = 1.34 + i0.1$
  5. Slab 5: $t_5 = 40\,nm$, $n_5 = 2.48$

3.Air (before the first layer and after the last layer): refractive index $n_{air} = 1$.

Key Equations:

•**Propagation matrix P_i**:

$$P_i = \begin{pmatrix} \exp(-in_ik_0t_i) & 0 \\ 0 & \exp(in_ik_0t_i) \end{pmatrix}$$

where $k0 = \frac{2\pi}{\lambda_0}$ is the wave number in a vacuum.

•**Transition matrix $T_{i-1,i}$**:

$$T_{i-1,i} = \frac{1}{2}\begin{pmatrix} 1 + \frac{\eta_{i-1}}{\eta_i} & 1 - \frac{\eta_{i-1}}{\eta_i} \\ 1 - \frac{\eta_{i-1}}{\eta_i} & 1 + \frac{\eta_{i-1}}{\eta_i} \end{pmatrix}$$

where η_i is the characteristic impedance, proportional to the inverse of the refractive index $\eta_i = \frac{1}{n_i}$.

•The final transfer matrix is:

$$M_{final} = T_{0,1}P_1T_{1,2}P_2 \ldots P_NT_{N,air}$$

where $k0 = \frac{2\pi}{\lambda_0}$ is the wave number in a vacuum.

•**Reflection and transmission coefficients:**

$$t = \frac{1}{M_{final}(1,1)}$$

$$r = \frac{M_{final}(2,1)}{M_{final}(1,1)}$$

Reflectance $R = |r|^2$, Transmittance $T = |t|^2$.

Step-by-step procedure:

1. Calculate the wave number $\boldsymbol{k_0}$:

$$k_0 = \frac{2\pi}{\lambda} = \frac{2\pi}{400nm} \approx 1.5708 \times 10^7 rad/m$$

2. Calculate propagation matrices $P_1, P_2, \ldots, P_5$ using the thicknesses and refractive indices of the slabs

3. Calculate transition matrices between each slab and between the final slab and air.

4 .Multiply all the matrices sequentially to get M_{final}

5. Extract reflection and transmission coefficients r and t, and compute the reflectance R and transmittance T.

I will now implement this process to find the reflectance and transmittance.

(c) LLM

```
import numpy as np

# Given parameters
wavelength = 400e-9  # 400 nm
k0 = 2 * np.pi / wavelength  # Wave number in vacuum

# Slab parameters: thickness (in meters) and complex refractive index (n)
slabs = [
    {"thickness": 20e-9, "n": 1.22},
    {"thickness": 25e-9, "n": 2.1 + 1j * 0.08},
    {"thickness": 30e-9, "n": 0.54 + 1j * 1.82},
    {"thickness": 20e-9, "n": 1.34 + 1j * 0.1},
    {"thickness": 40e-9, "n": 2.48},]

# Air refractive index
n_air = 1

# Function to calculate propagation matrix
def propagation_matrix(n, t, k0):
    phase_shift = np.exp(-1j * n * k0 * t)
    return np.array([[phase_shift, 0], [0, np.conj(phase_shift)]])

# Function to calculate transition matrix
def transition_matrix(n1, n2):
    eta1 = 1 / n1
    eta2 = 1 / n2
    t_matrix = 0.5 * np.array([
        [1 + eta1 / eta2, 1 - eta1 / eta2],
        [1 - eta1 / eta2, 1 + eta1 / eta2]])
    return t_matrix

# Initial transition matrix between air and slab 1
M = transition_matrix(n_air, slabs[0]["n"])

# Multiply through all layers
for i, slab in enumerate(slabs):
    # Propagation matrix for the current layer
    P = propagation_matrix(slab["n"], slab["thickness"], k0)
    # Add the propagation matrix for this layer
    M = M @ P
    # If not the last slab, include the transition to the next slab
    if i < len(slabs) - 1:
        M = M @ transition_matrix(slab["n"], slabs[i + 1]["n"])

# Final transition matrix between the last slab and air
M = M @ transition_matrix(slabs[-1]["n"], n_air)

# Calculate reflection and transmission coefficients
t = 1 / M[0, 0]
r = M[1, 0] / M[0, 0]

# Calculate reflectance and transmittance
R = abs(r)**2
T = abs(t)**2

R, T
```

(d) LLM

The calculated reflectance R is approximately 0.327, and the transmittance T is approximately 0.207 for the given multilayer structure with a plane wave of 400 nm wavelength incident perpendicularly in air.

FIG. S6. Results of failed one-shot CoT prompting for a five-layer structure. (a) The user's input prompt. (b) The reasoning process generated by the LLM. The LLM accurately followed the provided information to produce reasoning steps and equations. (c) The generated code for forward prediction of the five-layer structure contains misinterpretations, such as incorrectly applying conjugation to the phase term when defining the propagation matrix. (d) The results produced by the LLM. The correct groundtruth values are $R = 0.1998$ and $T = 0.1852$.

(a) **User**

Provide documentation on the transfer matrix method (TMM).

1. General description of TMM

Question: When a plane wave with a wavelength of 400 nm is incident perpendicularly, what are the reflectance and transmittance of the following structure?

Structure: Air/Slab 1/Slab 2/Air
- Slab 1: thickness 50 nm, refractive index 2.18
- Slab 2: thickness 100 nm, refractive index 0.14 + 1i*1.8

(b) **LLM**

To calculate the reflectance R and transmittance T of the given multilayer structure, we'll apply the transfer matrix method (TMM) as described in your formulation. Here's how we can break down the problem step by step:

Step 1: Define Key Parameters

Wavelength of incident light λ=400 nm

Wave number $k0 = \frac{2\pi}{\lambda}$

Slab 1: thickness t_1=50 nm, refractive index n_1=2.18

Slab 2: thickness t_2=100 nm, refractive index n_2=0.14+i·1.8

The air (incident medium and final medium) has a refractive index n_0=1.

Step 2: Construct the Propagation Matrices

For each layer, the propagation matrix P_i describes the phase accumulated as light travels through the material. The general form is:

$$P_i = \begin{bmatrix} e^{-in_ik_0t_i} & 0 \\ 0 & e^{in_ik_0t_i} \end{bmatrix}$$

Propagation Matrix for Slab 1:

$$P_1 = \begin{bmatrix} e^{-in_1k_0t_1} & 0 \\ 0 & e^{in_1k_0t_1} \end{bmatrix}$$

Propagation Matrix for Slab 2:

$$P_2 = \begin{bmatrix} e^{-in_2k_0t_2} & 0 \\ 0 & e^{in_2k_0t_2} \end{bmatrix}$$

Step 3: Construct the Transition Matrices

The transition matrix $T_{i-1,i}$ describes the transition between two materials (i.e., from layer $i-1$ to layer i) and is given by:

$$T = \frac{1}{2}\begin{bmatrix} 1+\frac{\eta_{i-1}}{\eta_i} & 1-\frac{\eta_{i-1}}{\eta_i} \\ 1-\frac{\eta_{i-1}}{\eta_i} & 1+\frac{\eta_{i-1}}{\eta_i} \end{bmatrix}$$

where $\eta_i = \frac{1}{n_i}$ is the characteristic impedance.

Transition Matrix from Air to Slab 1: Z

$$T_{0,1} = \frac{1}{2}\begin{bmatrix} 1+\frac{\eta_0}{\eta_1} & 1-\frac{\eta_0}{\eta_1} \\ 1-\frac{\eta_0}{\eta_1} & 1+\frac{\eta_0}{\eta_1} \end{bmatrix}$$

where $\eta_0 = 1$ (for air) and $\eta_1 = \frac{1}{n_1}$.

Transition Matrix from Slab 1 to Slab 2:

$$T_{1,2} = \frac{1}{2}\begin{bmatrix} 1+\frac{\eta_1}{\eta_2} & 1-\frac{\eta_1}{\eta_2} \\ 1-\frac{\eta_1}{\eta_2} & 1+\frac{\eta_1}{\eta_2} \end{bmatrix}$$

Transition Matrix from Slab 2 to Air:

$$T_{2,air} = \frac{1}{2}\begin{bmatrix} 1+\frac{\eta_2}{\eta_0} & 1-\frac{\eta_2}{\eta_0} \\ 1-\frac{\eta_2}{\eta_0} & 1+\frac{\eta_2}{\eta_0} \end{bmatrix}$$

Step 4: Multiply the Matrices to Find the Final Transfer Matrix M_{final}

The overall transfer matrix is the product of all the transition and propagation matrices:

$$M_{final} = T_{0,1}P_1T_{1,2}P_2T_{2,air}$$

Step 5: Calculate the Reflection and Transmission Coefficients

Once we have M_{final}, the reflection and transmission coefficients r and t can be computed using:

$$t = \frac{1}{M_{final}(1,1)}$$

$$r = \frac{M_{final}(2,1)}{M_{final}(1,1)}$$

Step 6: Compute the Reflectance and Transmittance

Finally, the reflectance R and transmittance T are given by:

$$R = |r|^2$$

$$T = |t|^2$$

Let me calculate the required propagation and transition matrices and compute R and T.

(c) LLM

```
import numpy as np

# Constants
wavelength = 400e-9  # 400 nm in meters
k0 = 2 * np.pi / wavelength  # Wave number

# Layer properties
n1 = 2.18  # Refractive index of slab 1
n2 = 0.14 + 1j * 1.8  # Refractive index of slab 2
t1 = 50e-9  # Thickness of slab 1 in meters
t2 = 100e-9  # Thickness of slab 2 in meters
n0 = 1  # Refractive index of air (both sides)

# Impedances
eta0 = 1 / n0
eta1 = 1 / n1
eta2 = 1 / n2

# Propagation matrices
P1 = np.array([[np.exp(-1j * n1 * k0 * t1), 0],
              [0, np.exp(1j * n1 * k0 * t1)]])

P2 = np.array([[np.exp(-1j * n2 * k0 * t2), 0],
              [0, np.exp(1j * n2 * k0 * t2)]])

# Transition matrices
T01 = 0.5 * np.array([[1 + eta0 / eta1, 1 - eta0 / eta1],
                      [1 - eta0 / eta1, 1 + eta0 / eta1]])

T12 = 0.5 * np.array([[1 + eta1 / eta2, 1 - eta1 / eta2],
                      [1 - eta1 / eta2, 1 + eta1 / eta2]])

T2_air = 0.5 * np.array([[1 + eta2 / eta0, 1 - eta2 / eta0],
                         [1 - eta2 / eta0, 1 + eta2 / eta0]])

# Final transfer matrix
M_final = np.dot(np.dot(np.dot(np.dot(T01, P1), T12), P2), T2_air)

# Reflection and transmission coefficients
t = 1 / M_final[0, 0]
r = M_final[1, 0] / M_final[0, 0]

# Reflectance and Transmittance
R = np.abs(r)**2
T = np.abs(t)**2

R, T
```

(d) LLM

The reflectance (R) and transmittance (T) of the given structure are approximately:

- Reflectance: $R = 0.908$
- Transmittance: $T = 0.0061$

This means that about 90.8% of the incident light is reflected, while only about 0.61% is transmitted through the multilayer structure.

FIG. S7. Results of successful one-shot CoT prompting for a double-layer structure. (a) The user's input prompt. (b) The reasoning process generated by the LLM. The LLM accurately produced reasoning steps and equations based on the provided information. (c) The generated code for forward prediction of the double-layer structure. (d) The results from the LLM align with the groundtruth values: $R = 0.9079$ and $T = 0.0061$.

(a)

User

Provide documentation on the transfer matrix method (TMM).
1. General description of TMM
2. Specific examples of TMM
   a. Single-layer structure example
   b. Triple-layer structure example

Question: When a plane wave with a wavelength of 400 nm is incident perpendicularly, what are the reflectance and transmittance of the following structure?

Structure: Air/Slab 1/Slab 2/.../Slab 9/Slab 10/Air
- Slab 1: thickness 20 nm, refractive index 1.438
- Slab 2: thickness 10 nm, refractive index 2.3 + 1i*0.03
- Slab 3: thickness 25 nm, refractive index 1.8
- Slab 4: thickness 30 nm, refractive index 0.14 + 1i*2.98
- Slab 5: thickness 30 nm, refractive index 2.71
- Slab 6: thickness 20 nm, refractive index 1.768 + 1i*0.12
- Slab 7: thickness 25 nm, refractive index 2.2
- Slab 8: thickness 50 nm, refractive index 1.41
- Slab 9: thickness 30 nm, refractive index 0.54 + 1i*1.82
- Slab 10: thickness 25 nm, refractive index 2.43

(b)

LLM

```
import numpy as np

# Constants
wavelength = 400e-9  # Wavelength in meters
k0 = 2 * np.pi / wavelength  # Wavenumber

# Define layer properties: [thickness (m), refractive index]
layers = [
    (20e-9, 1.438),
    (10e-9, 2.3 + 1j * 0.03),
    (25e-9, 1.8),
    (30e-9, 0.14 + 1j * 2.98),
    (30e-9, 2.71),
    (20e-9, 1.768 + 1j * 0.12),
    (25e-9, 2.2),
    (50e-9, 1.41),
    (30e-9, 0.54 + 1j * 1.82),
    (25e-9, 2.43)
]

# Air properties
n_air = 1  # Refractive index of air
eta0 = 377  # Characteristic impedance of free space

# Function to calculate the propagation matrix for each layer
def propagation_matrix(n, thickness):
    phase = n * k0 * thickness
    return np.array([
        [np.exp(-1j * phase), 0],
        [0, np.exp(1j * phase)]
    ])

# Function to calculate the transition matrix between two layers
def transition_matrix(eta1, eta2):
    return 0.5 * np.array([
        [1 + eta1 / eta2, 1 - eta1 / eta2],
        [1 - eta1 / eta2, 1 + eta1 / eta2]
    ])
```

LLM

```
    # Function to calculate the characteristic impedance for a layer
def characteristic_impedance(n):
    return eta0 / n

# Initialize total transfer matrix to identity matrix
M_total = np.eye(2, dtype=complex)

# Calculate the transfer matrix for the entire structure
for i in range(len(layers)):
    thickness, n = layers[i]
    eta_i = characteristic_impedance(n)

    if i == 0:
        # Transition matrix for air to first layer
        M_total = transition_matrix(characteristic_impedance(n_air), eta_i) @ M_total

    # Propagation matrix for the current layer
    P_i = propagation_matrix(n, thickness)

    # If it's not the last layer, calculate the transition to the next layer
    if i < len(layers) - 1:
        eta_next = characteristic_impedance(layers[i + 1][1])
        T_i_next = transition_matrix(eta_i, eta_next)
        M_total = T_i_next @ P_i @ M_total
    else:
        # Transition matrix for the last layer to air
        M_total = transition_matrix(eta_i, characteristic_impedance(n_air)) @ P_i @ M_total

# Calculate reflection and transmission coefficients
t = 1 / M_total[0, 0]
r = M_total[1, 0] / M_total[0, 0]

# Reflectance and transmittance
R = np.abs(r)**2
T = np.abs(t)**2

R, T
```

(c) LLM

The reflectance (R) and transmittance (T) for the given structure at a wavelength of 400 nm are:

- Reflectance (R): 0.349
- Transmittance (T): 0.041

This means that approximately 34.9% of the incident light is reflected, while 4.1% is transmitted through the structure.

FIG. S8. Results of failed few-shot CoT prompting for a 10-layer structure. (a) The code generated by the LLM contains an error in the matrix multiplication order, leading to an incorrect reflectance prediction, although the transmittance prediction is correct. (b) The final prediction from executing the code. The groundtruth values are $R = 0.7784$ and $T = 0.0412$.

(a)

User

Provide documentation on the transfer matrix method (TMM).
1. General description of TMM
2. Specific examples of TMM
   a. Single-layer structure example
   b. Triple-layer structure example

Question: When a plane wave with a wavelength of 400 nm is incident perpendicularly, what are the reflectance and transmittance of the following structure?

Structure: Air/Slab 1/Slab 2/.../Slab 9/Slab 10/Air
- Slab 1: thickness 20 nm, refractive index 1.438
- Slab 2: thickness 10 nm, refractive index 2.3 + 1i*0.03
- Slab 3: thickness 25 nm, refractive index 1.8
- Slab 4: thickness 30 nm, refractive index 0.14 + 1i*2.98
- Slab 5: thickness 30 nm, refractive index 2.71
- Slab 6: thickness 20 nm, refractive index 1.768 + 1i*0.12
- Slab 7: thickness 25 nm, refractive index 2.2
- Slab 8: thickness 50 nm, refractive index 1.41
- Slab 9: thickness 30 nm, refractive index 0.54 + 1i*1.82
- Slab 10: thickness 25 nm, refractive index 2.43

(b)

LLM

To calculate the reflectance and transmittance of the 10-layered structure with the provided parameters, we can follow the same Transfer Matrix Method (TMM) approach described earlier. Here's how we break down the process:

Given Parameters:

1.Wavelength (λ): 400 nm

2.Air: $n_0 = 1, \eta_0 = \eta_{air}$

3.Layer Refractive Indices and Thicknesses:
1. Slab 1: $n_1 = 1.438, t_1 = 20\,nm$
2. Slab 2: $n_2 = 2.3 + 1i \times 0.03, t_2 = 10\,nm$
3. Slab 3: $n_3 = 1.8, t_3 = 25\,nm$
4. Slab 4: $n_4 = 0.14 + 1i \times 2.98, t_4 = 30\,nm$
5. Slab 5: $n_5 = 2.71, t_5 = 30\,nm$
6. Slab 6: $n_6 = 1.768 + 1i \times 0.12, t_6 = 20\,nm$
7. Slab 7: $n_7 = 2.2, t_7 = 25\,nm$
8. Slab 8: $n_8 = 1.41, t_8 = 50\,nm$
9. Slab 9: $n_9 = 0.54 + 1i \times 1.82, t_9 = 30\,nm$
10. Slab 10: $n_{10} = 2.43, t_{10} = 25\,nm$

Steps to Solution:

1. Calculate Wavenumber k_0:

$$k_0 = \frac{2\pi}{\lambda} = 400 \times 10^{-9} = 1.5708 \times 10^{7} m^{-1}$$

2. Propagation Matrices P_i: The propagation matrix for each layer i is given by:

$$P_i = \begin{bmatrix} \exp(-i n_i k_0 t_i) & 0 \\ 0 & \exp(i n_i k_0 t_i) \end{bmatrix}$$

where n_i is the refractive index, and t_i is the thickness of the ith slab.

3. Transition Matrices $T_{i-1,i}$: The transition matrix between layers $i-1$ and i:

$$T_{i-1,i} = \frac{1}{2} \begin{bmatrix} 1 + \frac{\eta_{i-1}}{\eta_i} & 1 - \frac{\eta_{i-1}}{\eta_i} \\ 1 - \frac{\eta_{i-1}}{\eta_i} & 1 + \frac{\eta_{i-1}}{\eta_i} \end{bmatrix}$$

where η_i is the characteristic impedance of each layer.

4. Transfer Matrix M: For the 10-layer structure, the transfer matrix M_{final} is:

$$M_{final} = T_{0,1} P_1 T_{1,2} P_2 T_{2,3} P_3 T_{3,4} P_4 \ldots T_{9,10} P_{10} T_{10,air}$$

5. Reflection and Transmission Coefficients: Using the final transfer matrix M_{final}, the reflection (r) and transmission (t) coefficients are:

$$t = \frac{1}{M\_final(1,1)}$$

$$r = \frac{M\_final(2,1)}{M\_final(1,1)}$$

6. Reflectance (R) and Transmittance (T):

$$R = |r|^2$$

$$T = |t|^2$$

I will now proceed with the calculation of the reflectance R and transmittance T.

(c) LLM

```
import numpy as np

# Given values
wavelength = 400e-9  # 400 nm
k0 = 2 * np.pi / wavelength  # Wavenumber

# Slab properties: (n, t) where n is the refractive index and t is the thickness in meters
slabs = [
    (1.438, 20e-9),  # Slab 1
    (2.3 + 1j * 0.03, 10e-9),  # Slab 2
    (1.8, 25e-9),  # Slab 3
    (0.14 + 1j * 2.98, 30e-9),  # Slab 4
    (2.71, 30e-9),  # Slab 5
    (1.768 + 1j * 0.12, 20e-9),  # Slab 6
    (2.2, 25e-9),  # Slab 7
    (1.41, 50e-9),  # Slab 8
    (0.54 + 1j * 1.82, 30e-9),  # Slab 9
    (2.43, 25e-9),  # Slab 10
]

# Characteristic impedance of air
eta0 = 1  # Assuming η_0 = 1 for air

# Function to calculate propagation matrix P
def calc_propagation_matrix(n, t):
    phi = n * k0 * t
    return np.array([[np.exp(-1j * phi), 0], [0, np.exp(1j * phi)]])

# Function to calculate transition matrix T
def calc_transition_matrix(n1, n2):
    eta1 = eta0 / n1
    eta2 = eta0 / n2
    return 0.5 * np.array([[1 + eta1 / eta2, 1 - eta1 / eta2], [1 - eta1 / eta2, 1 + eta1 / eta2]])

# Initialize final transfer matrix as identity matrix
M_final = np.identity(2)

# Calculate the full transfer matrix
for i in range(len(slabs)):
    n_i, t_i = slabs[i]

    # For the first layer, transition from air to slab
    if i == 0:
        M_final = np.dot(calc_transition_matrix(1, n_i), calc_propagation_matrix(n_i, t_i))
    else:
        # Transition from slab i-1 to slab i and apply propagation matrix
        n_prev, _ = slabs[i-1]
        M_final = np.dot(M_final, calc_transition_matrix(n_prev, n_i))
        M_final = np.dot(M_final, calc_propagation_matrix(n_i, t_i))

# Add the final transition from the last slab to air
M_final = np.dot(M_final, calc_transition_matrix(slabs[-1][0], 1))

# Calculate reflection and transmission coefficients
t = 1 / M_final[0, 0]
r = M_final[1, 0] / M_final[0, 0]

# Reflectance and transmittance
R = np.abs(r)**2
T = np.abs(t)**2

R, T
```

(d) LLM

The reflectance (R) and transmittance (T) of the 10-layered structure when a plane wave with a wavelength of 400 nm is incident perpendicularly are:

- Reflectance $R = 0.778$
- Transmittance $T = 0.041$

FIG. S9. Results of successful few-shot CoT prompting for a 10-layer case. (a) The user's input prompt. (b) The LLM's reasoning process correctly followed the provided information to generate accurate reasoning steps and equations. (c) The code produced by the LLM. (d) The final results from executing the code align with the groundtruth values: $R = 0.7784$ and $T = 0.0412$.

```
def compute_reflectance_transmittance(layers, wavelengths):
    results = {}

    for wavelength in wavelengths:
        k_0 = 2 * np.pi / wavelength
        # Initialize M as identity matrix
        M = np.identity(2, dtype=complex)

        for i in range(len(layers)):
            t_i, n_i = layers[i]
            eta_i = eta_0 / n_i

            # Propagation matrix Pi for layer i
            P_i = np.array([
                [np.exp(-1j * n_i * k_0 * t_i), 0],
                [0, np.exp(1j * n_i * k_0 * t_i)]
            ], dtype=complex)

            # Transition matrix Ti between layer i and i+1
            if i == 0:
                # Transition from air to first layer
                eta_prev = eta_0
            else:
                _, n_prev = layers[i - 1]
                eta_prev = eta_0 / n_prev

            T_i = 0.5 * np.array([
                [1 + eta_prev / eta_i, 1 - eta_prev / eta_i],
                [1 - eta_prev / eta_i, 1 + eta_prev / eta_i]
            ], dtype=complex)

            # Multiply to form the characteristic matrix for the current layer
            C_i = T_i @ P_i
            M = M @ C_i

        # Final transition matrix from last layer to air
        n_last = layers[-1][1]
        eta_last = eta_0 / n_last
        T_last = 0.5 * np.array([
            [1 + eta_last / eta_0, 1 - eta_last / eta_0],
            [1 - eta_last / eta_0, 1 + eta_last / eta_0]
        ], dtype=complex)

        M_final = M @ T_last

        # Compute transmission (t) and reflection (r) coefficients
        t = 1 / M_final[0, 0]
        r = M_final[1, 0] / M_final[0, 0]

        # Reflectance and Transmittance
        R = np.abs(r) ** 2
        T = np.abs(t) ** 2

        results[wavelength] = (R, T)

    return results
```

FIG. S10. Code generated for the numerical simulation.

```
def objective_function(layers):
    error_sum = 0

    for wavelength in visible_range:
        # Get reflectance and transmittance using a pre-defined compute function
        R, T = compute_reflectance_transmittance(layers, [wavelength])[wavelength]

        # Target transmittance: high within passband, low outside passband
        if passband_range[0] <= wavelength <= passband_range[1]:
            target_transmittance = 1  # within passband range
        else:
            target_transmittance = 0  # outside passband range

        # Calculate squared error for this wavelength
        error_sum += (T - target_transmittance) ** 2

    # Return the cumulative error as the objective
    return error_sum
```

FIG. S11. Code generated for the objective function.

```
MAX_TOTAL_THICKNESS = 2e-6  # 2 microns in meters
MAX_NUM_LAYERS = 5

# Define realistic refractive indices for dielectric materials (approximate values)
# SiO2 (Silicon Dioxide), TiO2 (Titanium Dioxide), Al2O3 (Aluminum Oxide)
materials = [
    {'name': 'SiO2', 'refractive_index': 1.45},
    {'name': 'TiO2', 'refractive_index': 2.4},
    {'name': 'Al2O3', 'refractive_index': 1.77}
]
```

FIG. S12. Code generated for defining the structural configuration.

```
import numpy as np
import random
import matplotlib.pyplot as plt
from scipy.optimize import differential_evolution

MAX_TOTAL_THICKNESS = 2e-6  # 2 microns
MAX_NUM_LAYERS = 5
visible_range = np.linspace(400e-9, 700e-9, 100)  # 400–700 nm range
passband_range = (500e-9, 600e-9)  # 500–600 nm target passband
epsilon_0 = 8.854187817e-12
mu_0 = 4 * np.pi * 1e-7
eta_0 = np.sqrt(mu_0 / epsilon_0)

materials = [
    {'name': 'SiO2', 'refractive_index': 1.45},
    {'name': 'TiO2', 'refractive_index': 2.4},
    {'name': 'Al2O3', 'refractive_index': 1.77}
]

def compute_reflectance_transmittance(layers, wavelengths):

    results = {}

    for wavelength in wavelengths:
        k_0 = 2 * np.pi / wavelength
        M = np.identity(2, dtype=complex)

        for i in range(len(layers)):
            t_i, n_i = layers[i]
            eta_i = eta_0 / n_i

            P_i = np.array([
                [np.exp(-1j * n_i * k_0 * t_i), 0],
                [0, np.exp(1j * n_i * k_0 * t_i)]
            ], dtype=complex)

            if i == 0:
                eta_prev = eta_0
            else:
                _, n_prev = layers[i - 1]
                eta_prev = eta_0 / n_prev

            T_i = 0.5 * np.array([
                [1 + eta_prev / eta_i, 1 - eta_prev / eta_i],
                [1 - eta_prev / eta_i, 1 + eta_prev / eta_i]
            ], dtype=complex)

            C_i = T_i @ P_i
            M = M @ C_i

        n_last = layers[-1][1]
        eta_last = eta_0 / n_last
        T_last = 0.5 * np.array([
            [1 + eta_last / eta_0, 1 - eta_last / eta_0],
            [1 - eta_last / eta_0, 1 + eta_last / eta_0]
        ], dtype=complex)

        M_final = M @ T_last

        t = 1 / M_final[0, 0]
        r = M_final[1, 0] / M_final[0, 0]

        R = np.abs(r) ** 2
        T = np.abs(t) ** 2

        results[wavelength] = (R, T)

    return results
```

```
def objective_function(layer_params):
    layers = []
    total_thickness = 0
    for i in range(MAX_NUM_LAYERS):
        thickness = layer_params[i] * 1e-9  # Convert nm to meters
        n_i = materials[int(layer_params[MAX_NUM_LAYERS + i])]['refractive_index']
        layers.append((thickness, n_i))
        total_thickness += thickness
        if total_thickness > MAX_TOTAL_THICKNESS:
            return np.inf  # Penalty for exceeding total thickness

    error_sum = 0
    for wavelength in visible_range:
        R, T = compute_reflectance_transmittance(layers, [wavelength])[wavelength]
        target_transmittance = 1 if passband_range[0] <= wavelength <= passband_range[1] else 0
        error_sum += (T - target_transmittance) ** 2

    return error_sum

bounds = [(0, MAX_TOTAL_THICKNESS * 1e9 / MAX_NUM_LAYERS) for _ in range(MAX_NUM_LAYERS)]
+ [(0, len(materials) - 1) for _ in range(MAX_NUM_LAYERS)]

result = differential_evolution(objective_function, bounds, strategy='best1bin', popsize=15, maxiter=200,
mutation=(0.5, 1), recombination=0.7)

optimized_layers = []
for i in range(MAX_NUM_LAYERS):
    thickness_nm = result.x[i]
    material_idx = int(result.x[MAX_NUM_LAYERS + i])
    n_i = materials[material_idx]['refractive_index']
    optimized_layers.append((thickness_nm * 1e-9, n_i))

transmittance_values = []
for wavelength in visible_range:
    _, T = compute_reflectance_transmittance(optimized_layers, [wavelength])[wavelength]
    transmittance_values.append(T)\

# Plot the transmittance across the visible spectrum

target_transmittance = np.zeros([visible_range.shape[0]])
target_reflectance = np.zeros([visible_range.shape[0]])
for idx in range(visible_range.shape[0]):
    target_transmittance[idx] = 1 if passband_range[0] <= visible_range[idx] <= passband_range[1] else 0
    target_reflectance[idx] = 0 if passband_range[0] <= visible_range[idx] <= passband_range[1] else 1
plt.figure(figsize=(10, 5))
plt.plot(visible_range * 1e9, transmittance_values, 'bo-', label="Optimized T")
plt.plot(visible_range * 1e9, target_transmittance,'b--', label="Target T")
plt.plot(visible_range * 1e9, np.ones([visible_range.shape[0]])-transmittance_values, 'rv-', label="Optimized R")
plt.plot(visible_range * 1e9, target_reflectance,'r--', label="Target R")
plt.xlabel("Wavelength (nm)")
plt.ylabel("Transmittance")
plt.rc('font', size=20)
plt.rc('axes', labelsize=20)
plt.rc('xtick', labelsize=20)
plt.rc('ytick', labelsize=20)
plt.rc('legend', fontsize=15)

plt.legend()
plt.grid(True)
plt.show()
```

FIG. S13. The complete code was generated during the initial conversational loop. Modifications were applied to the visualization component.

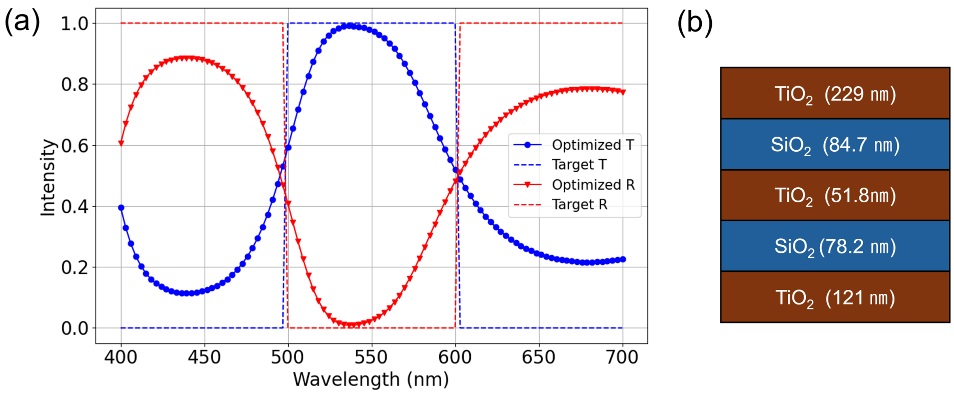

FIG. S14. A bandpass filter designed using the initial inverse design code shown in Figure S13. (a) The spectral response of the optimized structure. (b) The layout of the optimized structure consists of five alternating layers of high and low refractive index materials.

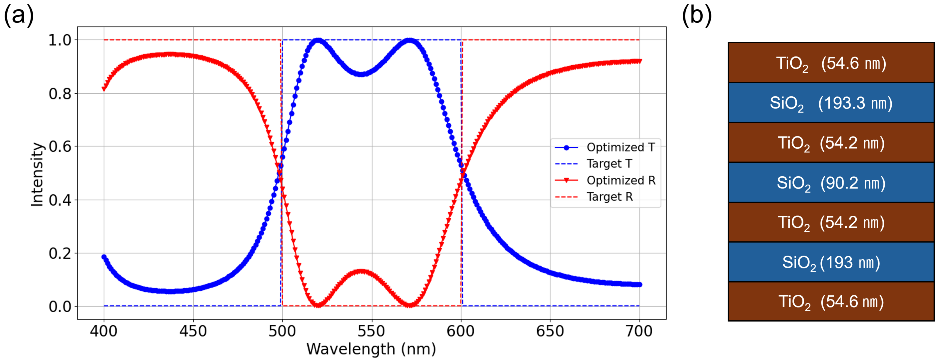

FIG. S15. A bandpass filter designed using the inverse design code modified with a single conversational feedback. (a) The spectral response of the optimized structure. (b) The optimized structure layout includes 7 alternating layers of TiO_2 and SiO_2.

```
import numpy as np
import random
import matplotlib.pyplot as plt
from scipy.optimize import differential_evolution

MAX_TOTAL_THICKNESS = 3e-6  # 3 microns
MAX_NUM_LAYERS = 20
visible_range = np.linspace(400e-9, 700e-9, 301)  # 400–700 nm range
passband_range = (500e-9, 600e-9)  # 500–600 nm target passband
epsilon_0 = 8.854187817e-12
mu_0 = 4 * np.pi * 1e-7
eta_0 = np.sqrt(mu_0 / epsilon_0)

materials = [
    {"name": "SiO2", "refractive_index": 1.45},   # Silicon Dioxide
    {"name": "TiO2", "refractive_index": 2.5},    # Titanium Dioxide
    {"name": "Al2O3", "refractive_index": 1.77},  # Aluminum Oxide
    {"name": "Si3N4", "refractive_index": 2.0},   # Silicon Nitride
    {"name": "HfO2", "refractive_index": 1.9}     # Hafnium Dioxide
]
def compute_reflectance_transmittance(layers, wavelengths):
    results = {}

    for wavelength in wavelengths:
        k_0 = 2 * np.pi / wavelength
        # Initialize M as identity matrix
        M = np.identity(2, dtype=complex)

        for i in range(len(layers)):
            t_i, n_i = layers[i]
            eta_i = eta_0 / n_i

                P_i = np.array([
                [np.exp(-1j * n_i * k_0 * t_i), 0],
                [0, np.exp(1j * n_i * k_0 * t_i)]
            ], dtype=complex)

            if i == 0:
                eta_prev = eta_0
            else:
                _, n_prev = layers[i - 1]
                eta_prev = eta_0 / n_prev

            T_i = 0.5 * np.array([
                [1 + eta_prev / eta_i, 1 - eta_prev / eta_i],
                [1 - eta_prev / eta_i, 1 + eta_prev / eta_i]
            ], dtype=complex)

            C_i = T_i @ P_i
            M = M @ C_i

        n_last = layers[-1][1]
        eta_last = eta_0 / n_last
        T_last = 0.5 * np.array([
            [1 + eta_last / eta_0, 1 - eta_last / eta_0],
            [1 - eta_last / eta_0, 1 + eta_last / eta_0]
        ], dtype=complex)

        M_final = M @ T_last

        t = 1 / M_final[0, 0]
        r = M_final[1, 0] / M_final[0, 0]

        # Reflectance and Transmittance
        R = np.abs(r) ** 2
        T = np.abs(t) ** 2

        results[wavelength] = (R, T)

    return results
```

```
def objective_function(layer_params):
    layers = []
    total_thickness = 0
    for i in range(MAX_NUM_LAYERS):
        thickness = layer_params[i] * 1e-9  # Convert nm to meters
        n_i = materials[int(layer_params[MAX_NUM_LAYERS + i])]['refractive_index']
        layers.append((thickness, n_i))
        total_thickness += thickness
        if total_thickness > MAX_TOTAL_THICKNESS:
            return np.inf  # Penalty for exceeding total thickness

    error_sum = 0
    for wavelength in visible_range:
        # Calculate R and T for this configuration and wavelength
        R, T = compute_reflectance_transmittance(layers, [wavelength])[wavelength]
        target_transmittance = 1 if passband_range[0] <= wavelength <= passband_range[1] else 0
        error_sum += (T - target_transmittance) ** 2

    return error_sum

bounds = [(0, MAX_TOTAL_THICKNESS * 1e9 / MAX_NUM_LAYERS) for _ in range(MAX_NUM_LAYERS)]
+ [(0, len(materials) - 1) for _ in range(MAX_NUM_LAYERS)]

result = differential_evolution(objective_function, bounds, strategy='best1bin', popsize=15, maxiter=300,
mutation=(0.5, 1), recombination=0.7)

optimized_layers = []
for i in range(MAX_NUM_LAYERS):
    thickness_nm = result.x[i]
    material_idx = int(result.x[MAX_NUM_LAYERS + i])
    n_i = materials[material_idx]['refractive_index']
    optimized_layers.append((thickness_nm * 1e-9, n_i))

transmittance_values = []
for wavelength in visible_range:
    _, T = compute_reflectance_transmittance(optimized_layers, [wavelength])[wavelength]
    transmittance_values.append(T)

# Plot the transmittance across the visible spectrum

target_transmittance = np.zeros([visible_range.shape[0]])
target_reflectance = np.zeros([visible_range.shape[0]])
for idx in range(visible_range.shape[0]):
    target_transmittance[idx] = 1 if passband_range[0] <= visible_range[idx] <= passband_range[1] else 0
    target_reflectance[idx] = 0 if passband_range[0] <= visible_range[idx] <= passband_range[1] else 1
plt.figure(figsize=(10, 5))
plt.plot(visible_range * 1e9, transmittance_values, 'bo-', label="Optimized T")
plt.plot(visible_range * 1e9, target_transmittance,'b--', label="Target T")
plt.plot(visible_range * 1e9, np.ones([visible_range.shape[0]])-transmittance_values, 'rv-', label="Optimized R")
plt.plot(visible_range * 1e9, target_reflectance,'r--', label="Target R")
plt.xlabel("Wavelength (nm)")
plt.ylabel("Transmittance")
plt.rc('font', size=20)
plt.rc('axes', labelsize=20)
plt.rc('xtick', labelsize=20)
plt.rc('ytick', labelsize=20)
plt.rc('legend', fontsize=15)

plt.legend()
plt.grid(True)
plt.show()
```

FIG. S16. The complete code was generated in the final conversational loop. Modifications were applied to the visualization component.
